# A Near Four-Decade Time Series Shows the Hawaiian Islands Have Been Browning Since the 1980s

**DOI:** 10.1007/s00267-022-01749-x

**Published:** 2022-11-22

**Authors:** Austin Madson, Monica Dimson, Lucas Berio Fortini, Kapua Kawelo, Tamara Ticktin, Matt Keir, Chunyu Dong, Zhimin Ma, David W. Beilman, Kelly Kay, Jonathan Pando Ocón, Erica Gallerani, Stephanie Pau, Thomas W. Gillespie

**Affiliations:** 1 Wyoming Geographic Information Science Center, University of Wyoming, Laramie, WY USA; 2 Department of Geography, University of California Los Angeles, Los Angeles, CA USA; 3 U.S. Geological Survey, Pacific Island Ecosystem Research Center, Honolulu, HI USA; 4 Army Natural Resources Program, Schofield Barracks, HI USA; 5 School of Life Sciences, University of Hawaiʻi at Mānoa, Honolulu, HI USA; 6 Department of Land and Natural Resources, Division of Forestry and Wildlife, Honolulu, HI USA; 7 School of Civil Engineering, Sun Yat-sen University, Zhuhai, China; 8 Department of Geography and Environment, University of Hawaiʻi at Mānoa, Honolulu, HI USA; 9 Department of Geography, Florida State University, Tallahassee, FL USA

**Keywords:** Advanced Very High Resolution Radiometer, Hawaiian Islands, Native ecosystems, Normalized Difference Vegetation Index, Palmer Drought Severity Index, Vegetation health

## Abstract

The Hawaiian Islands have been identified as a global biodiversity hotspot. We examine the Normalized Difference Vegetation Index (NDVI) using Climate Data Records products (0.05 × 0.05°) to identify significant differences in NDVI between neutral El Niño-Southern Oscillation years (1984, 2019) and significant long-term changes over the entire time series (1982–2019) for the Hawaiian Islands and six land cover classes. Overall, there has been a significant decline in NDVI (i.e., browning) across the Hawaiian Islands from 1982 to 2019 with the islands of Lāna’i and Hawai’i experiencing the greatest decreases in NDVI (≥44%). All land cover classes significantly decreased in NDVI for most months, especially during the wet season month of March. Native vegetation cover across all islands also experienced significant declines in NDVI, with the leeward, southwestern side of the island of Hawai’i experiencing the greatest declines. The long-term trends in the annual total precipitation and annual mean Palmer Drought Severity Index (PDSI) for 1982–2019 on the Hawaiian Islands show significant concurrent declines. Primarily positive correlations between the native ecosystem NDVI and precipitation imply that significant decreases in precipitation may exacerbate the decrease in NDVI of native ecosystems. NDVI-PDSI correlations were primarily negative on the windward side of the islands and positive on the leeward sides, suggesting a higher sensitivity to drought for leeward native ecosystems. Multi-decadal time series and spatially explicit data for native landscapes provide natural resource managers with long-term trends and monthly changes associated with vegetation health and stability.

## Introduction

The Hawaiian Islands have experienced high levels of habitat loss and degradation, and have experienced some of the highest extinction rates on the planet (Rolett and Diamond 2004; Caujapé-Castells et al. 2010; Triantis et al. 2010; Barton et al. 2021). Consequently, they contain some of the most endangered ecosystems in the world as well as 38% of all federally listed endangered and threatened plant species in the United States (Sakai et al. 2002; Pau et al. 2009; Fortini et al. 2013). Today, native ecosystems occur at high elevations and in fragmented patches in the lowlands (Juvik et al. 1998). Although much of Hawaii’s native lowland areas have been lost to development and agriculture, preservation of these remaining native ecosystems is both biologically and culturally important due to the high degree of endemism and rare species as well as the importance of many of these ecosystems to native Hawaiians (Cox 2000; Pascua et al. 2017; Knudson et al. 2018). Despite the high level of species and ecosystem endangerment, there is little comparative long-term data (i.e., decadal scale or longer) regarding the magnitude of land cover and ecosystem level changes across the Hawaiian Islands and the implications these have for conserving and managing native ecosystems (Lucas 2017; Barton et al. 2021).

There has been a rapid evolution in remote sensing of ecosystems, as exemplified by the number of scientific articles and reviews that use satellite imagery to assess land cover and land cover change, as well as techniques to model and monitor biodiversity and natural resource issues in a diverse array of ecosystems (Fraser et al. 2009; Secades et al. 2014; Gillespie et al. 2015). A number of common protocols and remote sensing techniques have been developed using rapid and standardized processing of large remote sensing datasets integrated with geographic information system (GIS) data (Hansen et al. 2013). Time series of vegetation indices and associated metrics (e.g., primary productivity and photosynthetic capacity) are an integrative way to measure ecosystem condition (Crabtree et al. 2009; Nemani et al. 2009). The Normalized Difference Vegetation Index (NDVI) and Enhanced Vegetation Index (EVI) are two of the most common vegetation indices used to study vegetation health and change (Pettorelli 2013). In particular, NDVI has been positively associated with photosynthetic activity, negatively associated with droughts, and has been hypothesized to quantify species richness or diversity within native ecosystems based on species-energy theories (Gillespie et al. 2008a; Pau et al. 2012; Pettorelli 2013; Xu et al. 2020). Thus, it should be possible to compare, map, and identify significant changes in photosynthetic activity, impacts of drought or drying, and species richness or diversity within different ecosystems between two time periods and across a full-time series.

The Advanced Very High Resolution Radiometer (AVHRR) sensor onboard National Oceanic and Atmospheric Administration (NOAA) satellites are the longest-running spaceborne earth resource sensor (Xu et al. 2020). AVHRR has provided daily weather and Earth observations since 1978 and imagery has been continuously collected since 1981. The recently released Climate Data Records (CDR) NDVI products from AVHRR at 0.05 × 0.05° spatial resolution include improved surface reflectance inputs, corrections for known errors in time, latitude, and longitude variables, and improvements in cloud masking and calibration monitoring (Vermote 2019). Results from AVHRR CDR and Moderate Resolution Imaging Spectroradiometer (MODIS) data show the consistency of the AVHRR CDR dataset, as both datasets have similar error (~10%) (Franch et al. 2017). Thus, AVHRR CDR data could provide a novel way to assess macro-ecosystem dynamics across a near four-decade time series.

Globally, there has been a significant increase in NDVI values or greenness due to climate warming, and this is especially true at high elevations and northern latitudes (Beck et al. 2011, Zhu et al. 2016, Xu et al. 2020). However, most global analyses do not provide information on remote islands like the Hawaiian Islands (Beck et al. 2011, Zhu et al. 2016). Within the Hawaiian Islands there is clear evidence of a warming trend from 1905 to 2017 (Kagawa-Viviani and Giambelluca 2020) and drying trends from 1905 to 2012 (Frazier and Giambelluca 2017; Clilverd et al. 2019).

There are a diverse array of habitat and landcover types on the Hawaiian Islands, ranging from low-elevation shrublands to high-elevation alpine regions, and a range of developed, agricultural, and protected areas. We would expect little to no change in NDVI or vegetation health within developed areas and either decreases in NDVI in agricultural areas due to the decline in pineapple and sugarcane production since the 1980s or no change as agricultural areas have gone fallow and been covered in non-native shrubland or forest (Suryanata 2002, Knudson et al. 2022). We would expect no or few significant changes in NDVI for native ecosystems, which are generally restricted to higher elevations and occur in protected areas created before the 1980s (Pettorelli et al. 2012, Protected Planet 2021). Whereas native alpine areas may be greening like other alpine regions worldwide (Zhu et al. 2016). However, extremes in temperature, decreases in precipitation, and increases in drought could result in long-term declines in NDVI or browning of the Hawaiian Islands and native ecosystems (Barbosa and Asner 2017, Frazier and Giambelluca 2017). To date, no studies have reported on long-term trends of NDVI for all of the Hawaiian Islands.

The systematic investigation and reliable prediction of the past and current status of ecosystems remains a grand challenge of environmental research and management. This research seeks to answer four questions based on a comprehensive geospatial understanding of land cover, ecosystem dynamics, and management: 1) Have there been significant annual and monthly changes in NDVI on the Hawaiian Islands from 1982 to 2019?, 2) Which land cover classes have changed the most on the Hawaiian Islands?, 3) Where have native ecosystems changed the most?, and 4) Are changes in native ecosystems associated with trends in precipitation and drought?

## Methods

### Study Area

**Fig. 1:**
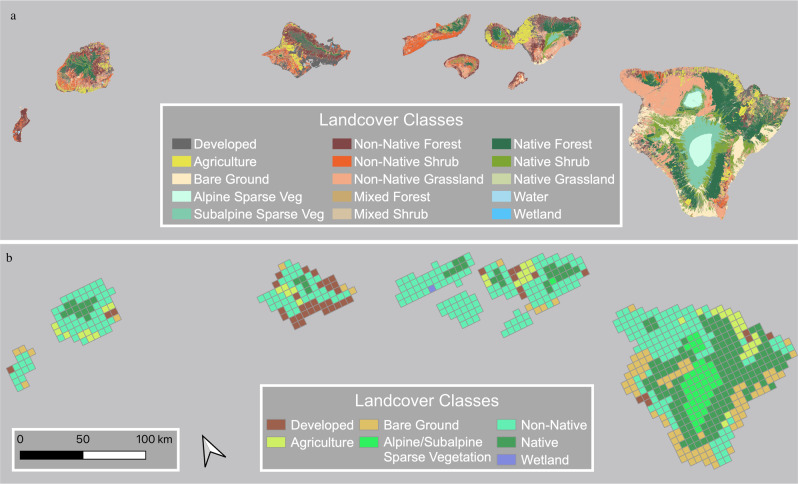
**a** Land cover classes at 30 m resolution for the Hawaiian Islands from the Carbon Assessment of Hawaii Land Cover Map (Jacobi et al. 2017) and **b** gridded map at a 0.05° spatial resolution depicting the seven aggregated land cover classes for the Hawaiian Islands

This research was conducted for the eight main Hawaiian Islands (Kaua’i, Niihau, O’ahu, Moloka’i, Maui, Lāna’i, Kaho’olawe, and the island of Hawai’i) located at the southeastern end of the archipelago. The Hawaiian Islands cover 16,634 km^2^ and are the result of an active hot spot in the middle of the Pacific Ocean. The islands range in age from 5.1 million years old (Kaua’i) to less than 0.5 million years old (island of Hawai’i). Elevations range from sea level to 4205 m on Mauna Kea, resulting in diverse climate zones and vegetation types (Jones and Bellaire 1937) (Fig. 1a).

The climate is predominantly controlled by orographic precipitation in which the north-easterly trade winds bring heavy rainfall to the windward (or wet) side of the volcanic mountains resulting in drier conditions on the leeward (or dry) side (Giambelluca et al. 2013). Mean annual precipitation is highly variable, with a rainy season occurring from November–April and a dry season from May–October (Frazier and Giambelluca 2017). Mean temperature ranges from 15.7 to 23.8 °C (Giambelluca et al. 2013).

### Remote Sensing Data

We acquired nearly four decades of gridded NDVI (0.05 × 0.05°) data at the daily temporal scale from the NOAA CDR archive. These daily NDVI arrays were derived from the AVHRR Surface Reflectance products as described in Franch et al. (2017) and Vermote (2019). The methodology described herein employed the recently released Version 5 CDR NDVI products as this version’s data utilized improved surface reflectance inputs as well as corrections for known errors in time, latitude, and longitude variables (Vermote 2019). Daily NDVI arrays were ingested into our workflow and clipped to the extent of the eight main islands of the Hawaiian Archipelago. We then created monthly time-series stacks of appropriately scaled CDR NDVI data by computing the median NDVI value for each cell in the grid for that particular month over the full temporal range in our dataset.

### Land Cover Classifications

We created a 0.05 × 0.05° (5.1 by 5.1 km) grid that corresponded to NOAA CDR pixel locations. We developed land cover classes for this grid based on the Carbon Assessment of Hawaii Land Cover Map (CAH) (Jacobi et al. 2017) (Fig. 1a). The reclassified land cover vector data were aggregated based on the majority land cover type within each 0.05 × 0.05° grid cell. This resulted in seven land cover classifications within the terrestrial study area: 1) Developed, 2) Agriculture, 3) Bare Ground (e.g., lava flows, sparse vegetation, and lowlands), 4) Alpine/Subalpine, 5) Non-Native (mixed and non-native forests, shrublands, and grasslands), 6) Native (native forests and shrublands), and 7) Wetlands (Fig. 1b). The Wetlands class only contained one pixel on the island of Moloka’i and was removed from the analysis. Thus, six land cover classes were aggregated into the NDVI grid cells (0.05 × 0.05°) and used to monitor vegetation health within the study area.

### Climate Data

Climate Hazards Group InfraRed Precipitation with Station data (CHIRPS) is a 40+ year quasi-global precipitation dataset. CHIRPS incorporates 0.05° resolution satellite imagery with in-situ station data to create gridded precipitation time series (1981 to present) for trend analysis and seasonal drought monitoring (Funk et al. 2015). The CHIRPS dataset is available in Google Earth Engine as an “ImageCollection” that provides total 5-day accumulated precipitation data at the global scale (Funk et al. 2015). The 5-day precipitation products were used to derive the total annual precipitation values used in this analysis.

Drought conditions were represented by monthly Palmer Drought Severity Index (PDSI) data products from the TerraClimate dataset. The TerraClimate PDSI products are global in scale, have a 0.5° spatial resolution, and include data from 1958 to 2018 (https://developers.google.com/earth-engine/datasets/catalog/IDAHO_EPSCOR_TERRACLIMATE) (Abatzoglou et al. 2018). PDSI uses temperature and precipitation data to estimate relative dryness as a standardized index that generally spans the range from −10 (dry) to +10 (wet) (Palmer 1965). We calculated the annual PDSI value for a given cell by using the median of the monthly PDSI values for that cell.

### Data Analysis

We used composite imagery to identify significant changes in NDVI from AVHRR at an aggregate level (all Hawaiian Islands, individual Hawaiian Islands, land cover classes, native ecosystems by island) as well as at the pixel level. We used the Oceanic Niño Index (ONI) to identify comparative years across the time series that occurred during similar neutral phases (−0.05 to 0.05) over the Hawaiian Islands (Trenberth and National Center for Atmospheric Research Staff 2020). The ONI is one of the most commonly used indices to define El Niño (<−0.05) and La Niña (>0.05) events and provides a quantitative measure of the neutral phases (−0.05 to 0.05) for which comparisons between two similar time periods can be undertaken (Guzmán et al. 2019). The calendar years 1984 and 2019 had similar neutral phase values (mean annual ONI −0.433 and 0.455 respectively) and were selected to identify significant changes in NDVI for all Hawaiian Islands, individual Hawaiian Islands, land cover classes, and native ecosystems by island. T-tests were used to identify significant differences in the annual median pixel values of NDVI between the 2 years 1984 and 2019 for the Hawaiian Islands, each individual island, land cover classes, and for native ecosystems by island.

For each month, we also used a Sen’s slope estimator and a linear least-squares regression on a cell-by-cell basis to derive the slope and the two-sided *p* value (*p*) from the associated null hypothesis test where time (in years) was regressed against the time-stacked monthly median NDVI values for that cell, respectively. We examined the percentage of cells with significant changes in NDVI for individual Hawaiian Islands by month based on AVHRR from 1982 to 2019 (nearly four decades). It is important to note that when examining percent change in NDVI, we only included pixels that had greater than 50% of their majority land cover class (Developed *n* = 9, Agriculture *n* = 20, Bare Ground *n* = 35, Alpine/Subalpine *n* = 46, Non-Native *n* = 180, Native *n* = 162) within a 5.1 km cell (e.g., the aggregation of the CAH land cover to our six land cover classes). It should be noted that 1982 was an El Niño year (mean ONI = 0.97) with ONI thresholds above 0.5 from April to December (Trenberth and National Center for Atmospheric Research Staff 2020). We also queried the Native class cells where *p* < 0.05 and determined the sign of the slope (increasing or decreasing) derive our final monthly vegetation greenness products. We then identified native cells that experienced the greatest declines in NDVI since 1982.

We estimated the long-term trends in annual total precipitation and annual mean Palmer Drought Severity Index (PDSI) for the period 1982–2019 in the Hawaiian Islands (Palmer 1965). To evaluate the effects of the long-term drying trends on native ecosystems or associated vegetation, we calculated the long-term precipitation anomaly (PA_t_) and cumulative precipitation anomaly (CPA_t_) using gridded precipitation data from CHIRPS. Because of the limited time range of the precipitation data, we used the first six years of the dataset (1982–1987) as the baseline period. PA_t_ and CPA_t_ were calculated as:

$${{{\mathrm{PA}}}}_t = \frac{{{{{\mathrm{Precipitation}}}}_t - {{{\mathrm{Precipitation}}}}_{{{{\mathrm{1982 - 1987}}}}}}}{{{{{\mathrm{Precipitation}}}}_{{{{\mathrm{1982 - 1987}}}}}}}$$

$${{{\mathrm{CPA}}}}_t = {{{\mathrm{PA}}}}_t + {{{\mathrm{PA}}}}_{{{{\mathrm{t - 1}}}}}$$

PA_t_ is the precipitation change in the year “*t*” relative to the baseline precipitation (1982–1987); *Precipitation*_*t*_ is the total precipitation in the year ‘*t*’ (mm) and *Precipitation*_*1982-1987*_ is the mean precipitation of the baseline period (1982 to 1987). Finally, the CPA_t_ is the sum of PA (relative change in precipitation) in the year “*t*” and “*t−1*”, showing how the relative change in precipitation accumulates throughout the approximately four decades of the time series (i.e., sum of percentage values).

As the Hawaiian Islands are dominated by a tropical maritime climate, with small seasonal temperature changes throughout the year, we calculated the median of the monthly NDVI as the annual NDVI value. Then we calculated the NDVI anomaly (VA) and the cumulative NDVI anomaly (CVA) as follows:

$${{{\mathrm{VA}}}}_t = \frac{{{{{\mathrm{NDVI}}}}_t - {{{\mathrm{NDVI}}}}_{{{{\mathrm{1982 - 1987}}}}}}}{{{{{\mathrm{NDVI}}}}_{{{{\mathrm{1982 - 1987}}}}}}}$$

$${{{\mathrm{CVA}}}}_t = {{{\mathrm{VA}}}}_t + {{{\mathrm{VA}}}}_{{{{\mathrm{t - 1}}}}}$$

where VA_t_ is the NDVI anomaly in the year “*t*” (relative departure from the mean NDVI between 1982 and 1987), NDVI_t_ is the NDVI value of each pixel in the year “*t*”, and NDVI_1982-1987_ is the median NDVI within the baseline period (1982–1987). CVA_t_ in the year “*t*” is the sum between the NDVI relative anomaly (VA_t_) in the year “*t*” and the year “*t* − *1*”.

We also estimated the long-term trends in annual mean Palmer Drought Severity Index (PDSI) for the period 1982–2019 in the Hawaiian Islands. We used the Sen’s slope estimator to determine the trends and applied the non-parametric Mann–Kendall test to identify the significance of these trends. A Pearson’s correlation was then utilized to investigate the relationship between the cumulative anomaly changes in precipitation and PDSI for native ecosystem NDVI.

## Results

### NDVI Changes on the Hawaiian Islands

**Fig. 2:**
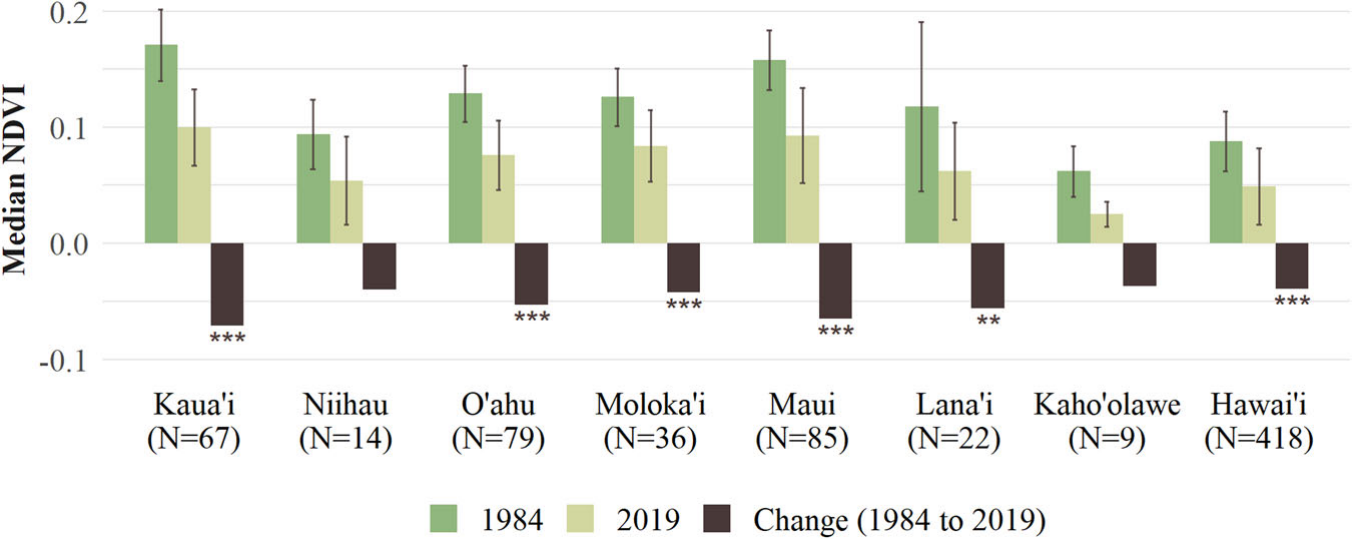
Number of pixels (*N*), median, standard deviation, change of NDVI from 1984 and 2019, and significant differences (95% confidence) based on a T-test for these 2 years (**p ≤ 0.01, ***p ≤ 0.001)

**Fig. 3:**
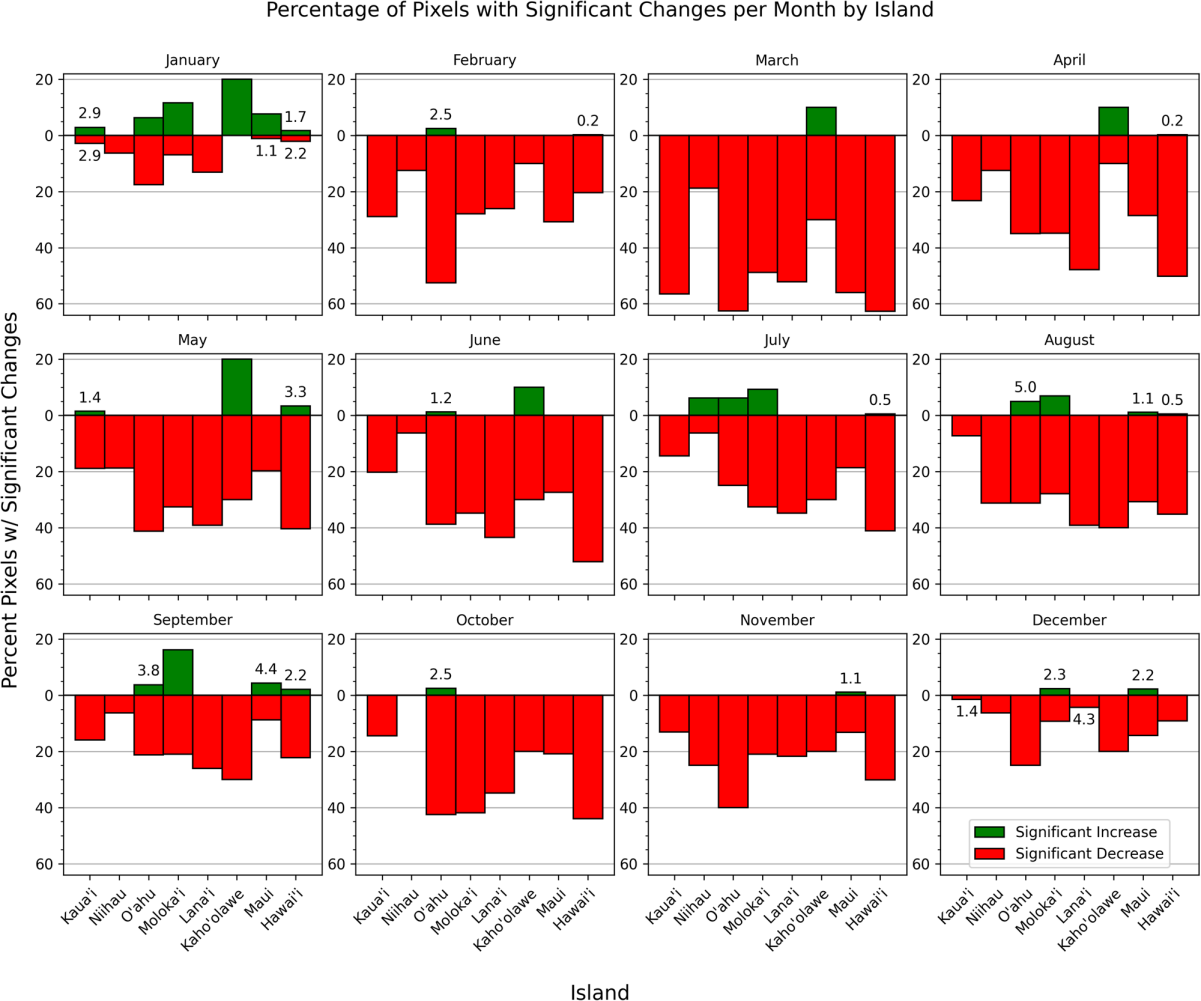
Percentage of pixels on the Hawaiian Islands that experienced a significant increase (green) or decrease (red) in median NDVI by month based on AVHRR (0.05°) from 1982 to 2019

Overall, there has been a significant decline in NDVI (i.e., browning) in the Hawaiian Islands from 1984 to 2019 (Median, T-test = 22.25, *p* < 0.001) (Table 1). A comparison of islands showed that there has been no significant overall change in NDVI for the small and low-lying islands of Niihau and Kaho’olawe (Fig. 2). However, Kaua’i, O’ahu, Moloka’i, Lāna’i, Maui, and Hawai’i all experienced significant declines in NDVI between 1984 and 2019. For all months except January, significant declines in NDVI occurred more frequently than increases, especially from February to November (Fig. 3, Online Resource 1).

**Table 1:** Summary statistics of annual normalized difference vegetation index (NDVI) using AVHRR imagery over the Hawaiian Islands during similar ENSO neutral phase conditions (1984 and 2019)

Year	Mean	St. Dev.	Median	Minimum	Maximum
1984	0.115	0.036	0.103	−0.098	0.725
2019	0.070	0.041	0.054	−0.100	0.793
Change	−0.045	−0.005	−0.049	−0.002	0.068

O’ahu, Lāna’i, Moloka’i, and Hawai’i experienced the greatest browning or decrease in NDVI from 1982 to 2019 (Fig. 3). There were relatively few significant changes in NDVI at the pixel level for the month of December, and most islands experienced significant increases in NDVI for the month of January. During the month of March, most islands experienced the largest percent of pixels with significant declines in NDVI.

### NDVI Change by Land Cover

**Fig. 4:**
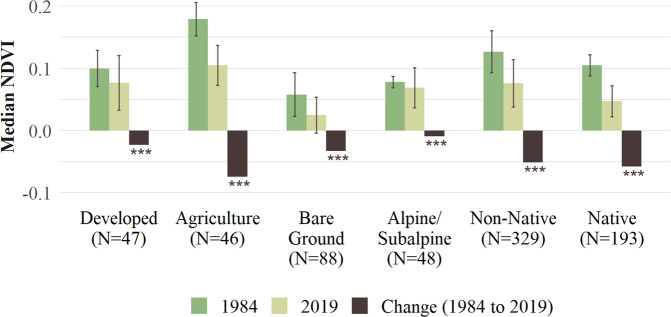
Land cover classes on the Hawaiian Islands, number of pixels, median, standard deviation, and median change in NDVI from 1984 and 2019, with significant differences based on a T-test (***p ≤ 0.001)

The Non-Native class (non-native and mixed non-native/native) covered the largest area (44%) on the Hawaiian Islands, followed by the Native class (26%) and the Bare Ground class (12%), while Developed (6%), Agriculture (6%), and Alpine/Subalpine (6%) classes covered similar areas on the Hawaiian Islands. The Agriculture class was the greenest, followed by the Non-Native class, and interestingly, the Developed and Native classes had similar median NDVI values (Fig. 4). All land cover classes experienced a decrease in NDVI values during the study period.

**Fig. 5:**
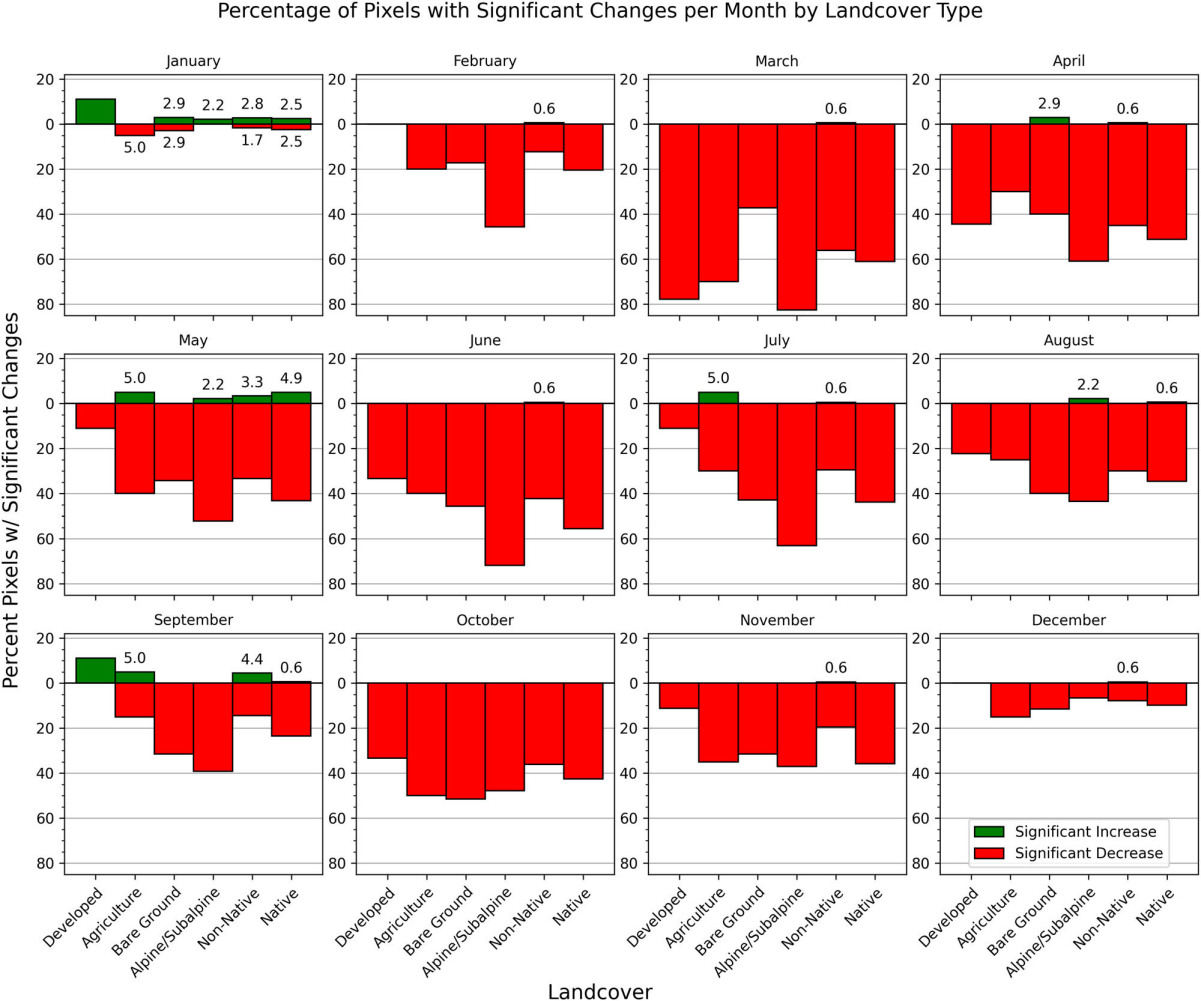
Percentage of pixels in each land cover class that experienced a significant increase (green) or decrease (red) in median NDVI by month based on AVHRR (0.05°) from 1982 to 2019. Analysis only included cells with over 50% of one aggregated Carbon Assessment of Hawaii land cover type

When monthly changes in NDVI were examined for pixels with over 50% of one aggregated CAH land cover type in a given AVHRR cell, significant decreases were more common than increases for most of the land cover classes and months (Fig. 5). Developed areas were the most stable of all land cover types and experienced a significant increase in NDVI for the months of May and September. The Agriculture class experienced significant increases in January and May and decreases for all other months. Bare Ground and Alpine/Subalpine classes all had a high percentage of pixels that significantly decreased in NDVI (except for January). Non-Native and Native classes showed similar monthly patterns, with both experiencing more significant declines in NDVI than increases for all months except January and September. The Native class experienced the third greatest proportional declines of all land cover classes, with over 40% of Native class cells experiencing significant declines in NDVI during the months of March, April, May, June, July, and October.

### NDVI Change of Native Vegetation

**Fig. 6:**
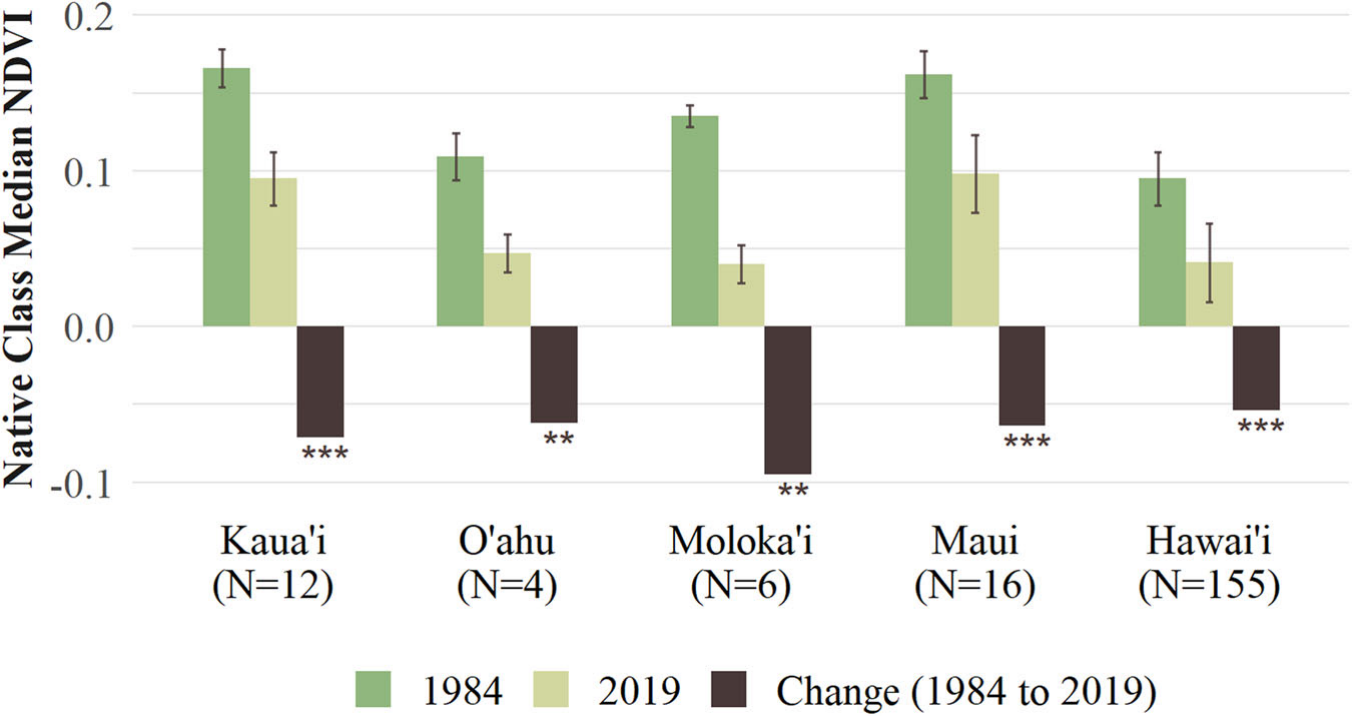
Description of native pixels on five Hawaiian Islands, including number of pixels (*N*), median and standard deviation of NDVI from 1984 and 2019, and change in NDVI, with significant differences based on a T-test (**p ≤ 0.01, ***p ≤ 0.001)

Native class cells were identified on the five largest islands (Kaua’i, O’ahu, Moloka’i, Maui, and Hawai’i). In 1984, Maui and Kaua’i had the highest median NDVI values for native ecosystems while Moloka’i, Hawai’i, and O’ahu had the lowest median NDVI values (Fig. 6).

By 2019, the median NDVI values decreased in native ecosystems across all five islands and the standard deviation of NDVI generally increased between the two time periods, suggesting increased variability in vegetation health over time.

**Fig. 7:**
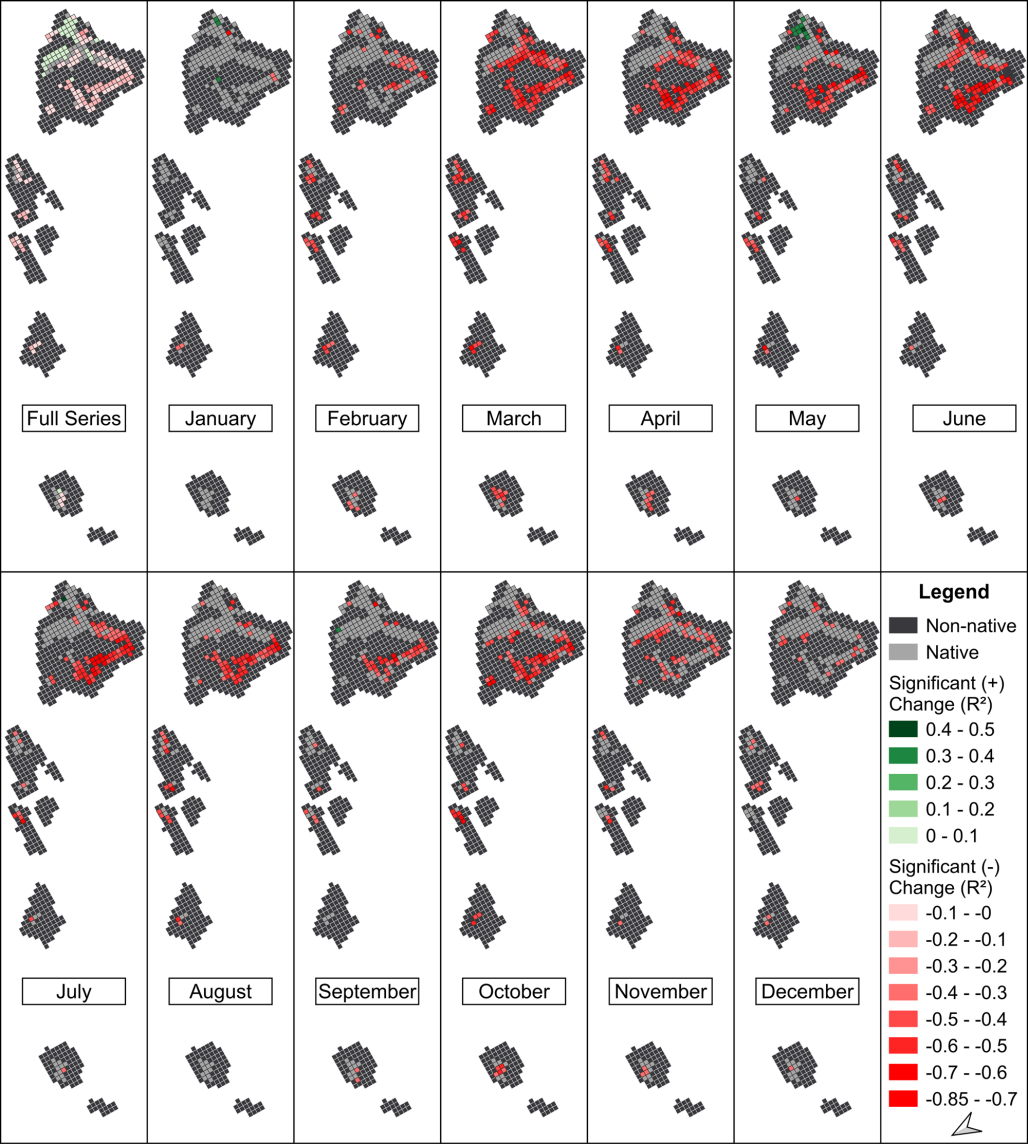
Significant changes in NDVI from a linear regression and Pearson correlation coefficient values (*r*) for the Native class across the full-time series (1982–2019). The “Full Series” subplot denotes changes over the entirety of the time series and the “Monthly” subplots denote changes over the full-time series for a given month. Red cells denote Native pixels with a significant negative change in median NDVI. Green cells denote Native pixels with a significant positive change in NDVI. Light-gray cells denote Native pixels without significant changes in NDVI. Dark-gray cells denote pixels with other land cover class types

Across the full-time series, native ecosystems on Maui and Kaua’i were the least variable. The northeastern side of Hawai’i was stable or experienced increases in NDVI while almost the entire southwestern side of Hawai’i experienced a significant decline in NDVI and contained the highest Pearson correlation coefficient (*r*) (Fig. 7: “Full Series”). When monthly results were examined for Hawai’i, the Pearson correlation coefficient values (*r*) increased significantly, with March to October having the greatest decrease in NDVI (especially on the southwestern side of the island). No increases in NDVI were observed in Native pixels on O’ahu, Moloka’i, Maui, or Kaua’i during any given month (Fig. 7: “Monthly”).

### Links between Precipitation/Drought and NDVI on Native Vegetation Health

**Fig. 8:**
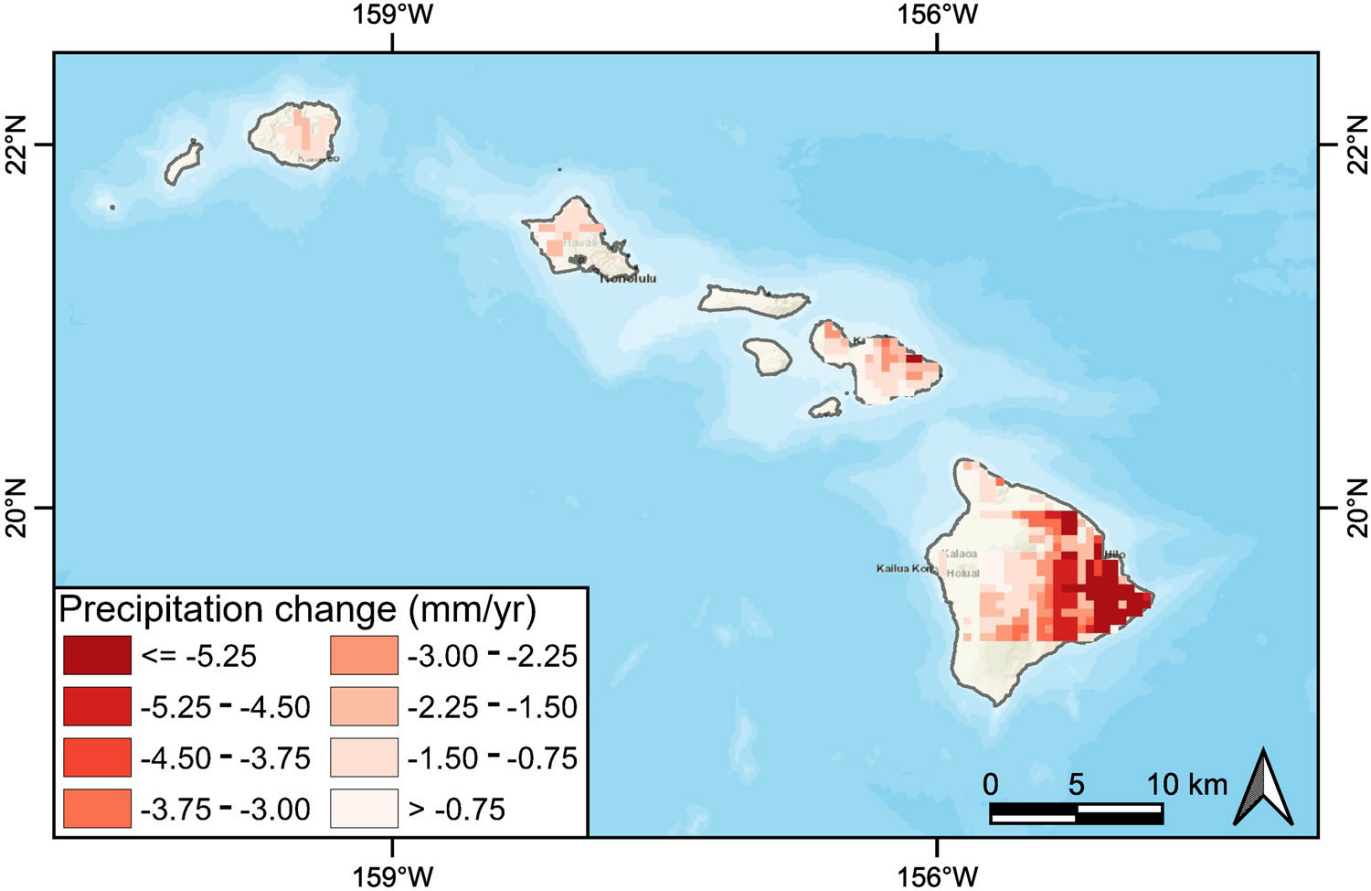
Spatial distribution of the trends in annual total precipitation change during the period 1982–2019. Trends were calculated using a Sen’s slope estimator. Only significant (*p* < 0.05) pixels are shown

**Fig. 9:**
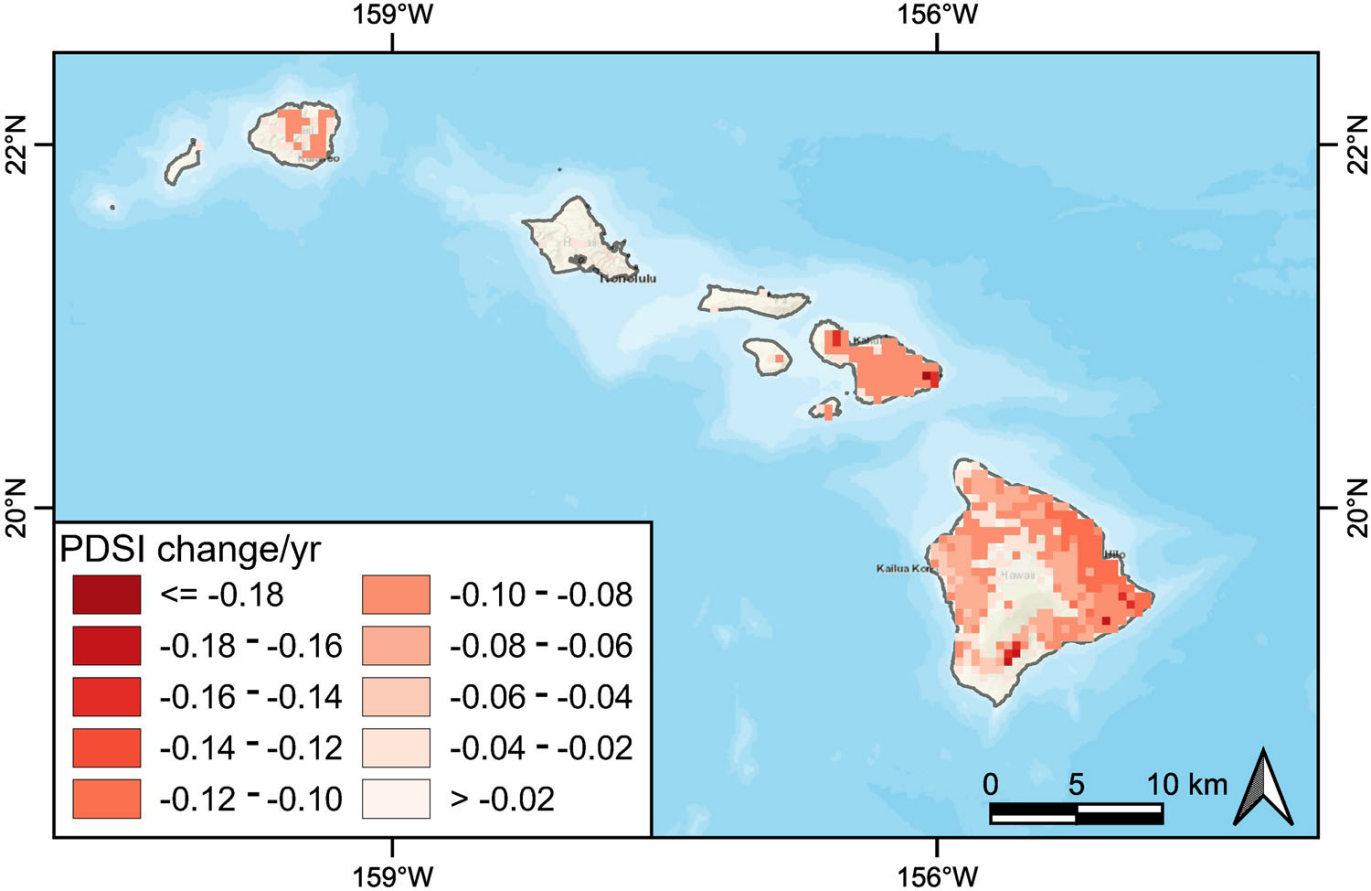
Spatial distribution of Palmer Drought Severity Index (PDSI) trends during the period 1982–2019. Trends were calculated using a Sen’s slope estimator. Only the significant (*p* < 0.05) pixels are shown

We estimated the long-term trends in annual total precipitation and annual mean PDSI for the period 1982–2019 on the Hawaiian Islands and used the Sen’s slope estimator to determine the trends along with the Mann-Kendall test to identify the significance of those trends. Precipitation (Fig. 8) and PDSI (Fig. 9) both show significant declines, and the decreases were particularly evident on the windward side of the island of Hawaii. The impact of drought, as evidenced by PDSI, was more widespread than precipitation, and this is especially true on the leeward side of Maui and Hawai’i and at lower elevations.

**Fig. 10:**
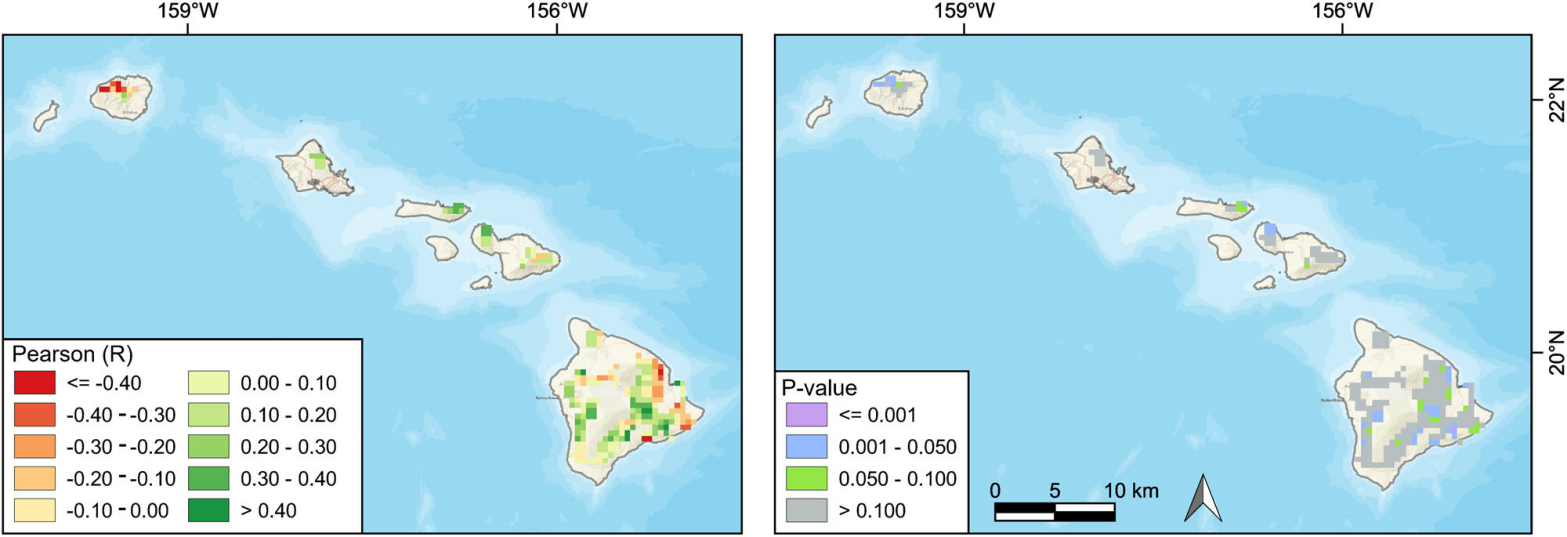
Spatial distribution of the Pearson’s correlation coefficients and *p* values between cumulative anomaly changes in precipitation and the native ecosystem NDVI

A Pearson’s correlation was then utilized to investigate the relationship between the cumulative anomaly changes in precipitation and native vegetation NDVI (Fig. 10). Correlations between the native vegetation NDVI and precipitation were primarily positive, suggesting that significant decreases in precipitation may exacerbate the decrease in NDVI of the native ecosystems over the time series.

**Fig. 11:**
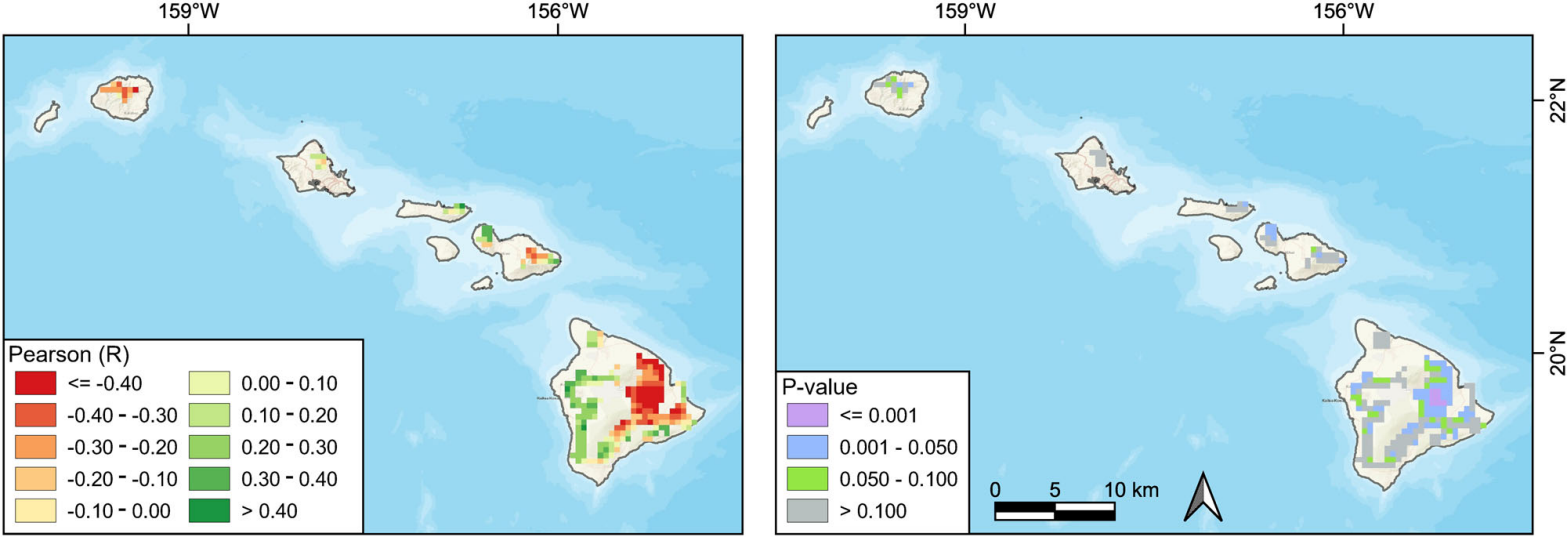
Spatial distribution of the Pearson’s correlation coefficients and *p* values between Palmer Drought Severity Index and native ecosystem NDVI from 1982 to 2019

We also used Pearson’s correlation to investigate the relationship between PDSI and the native vegetation NDVI during the period of 1982–2019. The NDVI-PDSI correlations were primarily negative on the windward side of the islands and positive on the leeward side (Fig. 11). This opposite response of NDVI to PDSI on the windward and leeward slopes may imply divergent limiting factors of the native plants on the two sides. Compared to the vegetation on the windward slope, the vegetation on the leeward side seems to be more vulnerable to the drying climate, and thus the decrease in NDVI is more obvious.

## Discussion

### Examining NDVI Changes on the Hawaiian Islands

Overall, NDVI (e.g., photosynthetic activity or vegetation health) on the Hawaiian Islands declined from 1982 to 2019. This is contrary to a global trend that has identified increases in NDVI since 1982 (Beck et al. 2011; Zhu et al. 2016). Time series results from the Hawaiian Islands appear similar to trends in the northern hemisphere tropics (Xu et al. 2020). Indeed, global breakpoints using BFAST models identified in Xu et al. (2020) are evident in the time series trends for native ecosystems (Online Resource 2: SI 1). Further, an examination of the NDVI times series for the “purest” CAH aggregated pixels for each landcover class shows a significant reduction in NDVI values that corresponds with a notable La Niña event in 1989 (Online Resource 2: SI 2). This information suggests that macro-trends in NDVI in the Hawaiian Islands track El Niño-Southern Oscillation (ENSO) and associated phenological patterns in the northern hemisphere tropics (e.g., Central America, North Africa, Asia) (Pau et al. 2010). This highlights the need to compare ENSO-neutral years and seasons when undertaking comparative change detection analyses (Guzmán et al. 2019). This also suggests that individual pixels that do not follow these macro-phenology trends (e.g., anomalies) may be impacted by other factors. These factors include long-term drying, land use management (e.g., agriculture), changes in species composition (e.g., non-native grasses), fire, and/or grazing.

The declines in NDVI for all islands and significant declines in NDVI over the time series for the largest six islands are surprising. All islands experienced a 33% or greater decline in NDVI between 1984 and 2019 (neutral phase years), especially the islands of Lāna’i (47% decline) and Hawai’i (44% decline). The greatest changes in NDVI occurred in March for all islands, which is the fourth wettest month on the Hawaiian Islands after November, December, and January. Declines in March could be related to lags in browning due to decreased precipitation, increased evapotranspiration during previous months, and/or the result of increased cloud cover (Pau et al. 2010).

### Examining NDVI Changes for Different Land Cover Classes

All land cover classes (>50% one land CAH cover type) have experienced significant declines in NDVI over the time series. Overall, developed areas experienced a significant decline in NDVI or photosynthetic activity. However, there was evidence of increased greenness in May and September, which corresponds to the beginning and the end of the dry season. Overall declines in NDVI may be related to the expansion of developed areas into former agricultural lands or an increase of impervious surfaces in urban and suburban areas over the time series (Lucas 2017; Creighton 2021). The Agriculture class covered the most area on Kaua’i, O’ahu, Maui, and eastern Hawai’i, and contained the highest NDVI values of all land cover classes. This fact is proof-positive that our AVHRR NDVI-based methods are robust as agricultural areas should contain very healthy vegetation. However, the decline of agriculture on the Hawaiian Islands has been well documented (Suryanata 2002; Knudson et al. 2022) and we also found significant declines in NDVI for the Agriculture class. These declines appear to correspond with decreased irrigation of fields over the time series and the colonization of fallow fields by invasive plants that have lower NDVI values than traditional crop types (e.g., sugar cane) (Knudson et al. 2022).

The Bare Ground class occurred primarily at low elevations in dry areas in Hawai’i (e.g., Kona, Volcanoes National Park) with the greatest decreases occurring in the summer (June–October), which generally corresponds to the dry season in Hawaii (May–October) (Frazier and Giambelluca 2017). Bare Ground had the lowest NDVI values but still showed evidence of declines in photosynthetic activity. Again, this shows that our AVHRR NDVI-based methods are robust as regions of bare ground should contain low NDVI values due to a lack of vegetation. The causes of these declines are complex and most likely related to land use management, moisture availability, grazing, and frequent fires which can be common even in low biomass environments (Lucas 2017; Creighton 2021).

Alpine/Subalpine classes were primarily located on the island of Hawai’i with only a single cell in Haleakala National Park, Maui. Globally, alpine areas have experienced warming and increases in greening (Zhu et al. 2016). Interestingly, the Alpine/Subalpine class proportionally experienced significant NDVI declines from February to July. Previous research has shown that a higher frequency of trade wind inversions at these high elevations has been detected on Maui, and these can lead to drying-related declines of subalpine plant species (Krushelnycky et al. 2013). However, since 2015 there is evidence of increased greenness in the Alpine/Subalpine areas above 2000 m in Hawai’i (Online Resource 2: SI 3), and this may be related to increased temperatures and associated plant colonization at higher elevations.

The Non-Native class, which we classified as either non-native and/or a mix of native/non-native plants, was the dominant land cover type (44%) within the study area and had the second highest (after Agriculture) NDVI values of the six land cover classes. These non-native ecosystems are heterogeneous in floristic composition but can generally be sub-classified as non-native grasslands (*Pennisetum setaceum*, *Pennisetum ciliare*, *Pennisetum clandestinum*), shrublands (*Schinus terebinthifolius*, *Leucaena leucocephala*), and forests (*Psidium cattleianum, Miconia calvescens*) (Gillespie et al. 2008b). Non-native ecosystems generally track native ecosystem phenology (Fig. 5) but did not decrease as much as native ecosystems. Non-Native classes appear to have higher biomass and higher variability in NDVI than the Native class over the time series (Asner et al. 2008). Many of the widespread non-natives lose their leaves during drought and this, along with fire, may lead to higher NDVI variability in the Non-Native class.

### Examining NDVI Changes of Native Vegetation

Native ecosystems on O’ahu (56%), Moloka’i (70%), and Hawai’i (57%) decreased the most in NDVI from 1984 to 2019. The native ecosystems on the southwestern (Kona) side of Hawai’i declined the most across the full time series and by month (March–October). This browning was also evident for western Hawai’i in MODIS imagery from 2002 to 2016 (Barbosa and Asner 2017). This region contains a mix of native mesic and wet forest and shrubland and has high cloud cover throughout the year. Indeed, this region is part of the cloud zone that contains a vast majority of Hawaii’s native biodiversity (Kagawa-Viviani and Giambelluca 2020). Within this region, there is no evidence of forest loss from 2000 to 2019 from a Landsat analysis at 30 m pixel resolution, and little evidence of fire in this region based on state and MODIS data, or Rapid Ohia Death (ROD) due to *Ceratocystis* spp. (Online Resource 2: SI 4a-e). Using 0.05° grids across Kaua’i, O’ahu, Moloka’i, Maui, and Hawai’i along with climate data from CHIRPS (a 40+ year precipitation dataset at 0.05° resolution) (Funk et al. 2015), we found significant long-term drying in this region. It appears that windward side native vegetation is more limited by radiation than precipitation given the relatively moist climate in those regions (Graham et al. 2003). Thus, increased solar radiation during drought might promote vegetation productivity on the windward slope. However, the relatively drier climate on the leeward side leads to a higher sensitivity of its native vegetation to drought, as indicated by the positive NDVI-PDSI correlations here. Indeed, Frazier and Giambelluca (2017) mapped long-term precipitation trends for the Hawaiian Islands and also identified long-term drying in this region, especially during the dry season (May–October seasons). Additionally, a 20-year analysis identified increases in drought on the Hawaiian Islands, and especially on the leeward side of Hawai’i (Leeper et al. 2022). The extent of the heavily impacted areas overlaps with declines in NDVI in the native ecosystems on the southwestern side Hawai’i. Repeat airborne lidar in 2007 and 2016 suggest a decrease in canopy height and volume in these drying areas on the island of Hawai’i (Barbosa and Asner 2017). Thus, the declines in NDVI or photosynthetic activity are most likely related to long-term drying, canopy volume loss, tree mortality, and possibly further impacted by the lack of regeneration due to grazing of goats and cattle (Barbosa and Asner 2017; Frazier and Giambelluca 2017; Lucas 2017).

NDVI is theoretically and empirically associated with photosynthetic activity, primary productivity, drought stress, and has been hypothesized to capture ~40% of species richness or diversity within native habitat types (Pettorelli 2013, Pau et al. 2012). Thus, it would appear that within native habitat types, primary productivity and possibly biodiversity have been declining in selected pixels from 1982 to 2019, and the impacts of drought or drying may be a primary cause (Xu et al. 2020). In the future, if the drying and warming of the climate on the leeward slope of the island of Hawai’i continue, native ecosystems may become increasingly vulnerable to fire and succumb to the expansion of invasive species.

### Applications of AVHRR for Ecosystem Monitoring

The long timespan of the AVHRR CDR data (~40 years) allows for historical analyses of land use and land cover change for regions across the world, as our Hawaiian analysis shows (Colwell and Hicks 1985; Xu et al. 2020). In this study, time series results can be used by natural resource managers to identify if landscapes, native ecosystems, or protected areas have 1) been stable across the time series (thus suggesting healthy vegetation and appropriate management strategies), or 2) experienced significant declines across the time series or monthly trends (suggesting vegetation health may be declining and the causes and appropriate management strategies need to be identified) (Alcaraz-Segura et al. 2009). For example, Kaʿūpūlehu on Hawai’i is a well-known tropical dry forest remnant site that has experienced a decline in NDVI since 1982 for 11 out of 12 months of the year with 6 of those months showing a significant decline (Online Resource 2: SI 5a) (Litton et al. 2006) despite highly successful restoration efforts (Libby et al. 2022). Low NDVI values at Kaʿūpūlehu directly track prolonged drought periods (1998–2003 and 2007–2014), and the overall decline is likely the result of the combination between a significant decrease in precipitation across the landscape over the past century (Frazier and Giambelluca 2017), the removal of non-native trees, and a slow loss of canopy trees that was accompanied by little regeneration prior to restoration efforts. Nonetheless, post-drought rebound of NDVI values also demonstrates the resilience of the restored forest, and over time can potentially be used to track the regenerating native canopy. Auwahi, on Maui, (Online Resource 2: SI 5b) is a well-known tropical dry forest that has been restored from pasture and has experienced a significant increase in NDVI as native canopy trees replace non-native grasses. Species population trend data for endangered and threatened species can be compiled and compared to trends of NDVI or ecosystem health (Craven et al. 2018, Libby et al. 2022). Indeed, trends in NDVI can also be compared to trends in bird populations, with most of the endangered bird species restricted to nine or fewer AVHRR CDR NDVI cells (Barton et al. 2021).

AVHRR CDR data also provide a rationale to further investigate landscape-level change detection using higher resolution data such as Landsat (30 m) and very high-resolution imagery (<3 m). Natural resources managers will not have control over the impacts of climatic changes or drought, but they may be able to prioritize and stabilize the health of native ecosystems or native/non-native mixed ecosystems via reduction in human-caused fire, grazing, or through the removal of non-native or invasive species that impact native vegetation extent and quality. Another potential management application for this study is the guidance of outplanting or transplanting of native plants in stable areas. Our NDVI-based analysis could potentially help guide plantings towards locations with the lowest NDVI decreases. Further, subsequent monitoring using these methods could help identify the links between outplanting success and monthly changes in NDVI.

### Study Limitations

There are a number of limitations that should be considered when applying AVHRR NDVI data for this kind of analysis (e.g., pixel size, cloud cover, and environmental impacts). The pixel sizes are large (i.e., 0.05 × 0.05°), and the corresponding assessments of land cover classes must match the coarse spatial resolution. This aggregation of land cover data provides for less detail in the class-by-class NDVI analysis. The extent and changes of cloud cover may impact monthly NDVI values (Pau et al. 2010), and this is especially true in regions of seasonally high cloud cover. Increased cloud cover is common on the higher elevation windward sides of the Hawaiian Islands (areas that contain the highest share of remnant native biodiversity) due to orographic uplift of moisture-laden air (Kagawa-Viviani and Giambelluca 2020). There are several other factors beyond precipitation and drought that may lead to changes in NDVI that were not implicitly considered in this analysis, e.g., monthly lags in native vegetation NDVI and climatic metrics, changes in fire regime, changes in land use and cover, and grazing. There is little comparative spatial data on the impacts of grazing by non-native herbivores such as goats, pigs, and cattle (Cabin et al. 2000). Thus, it is not currently possible to determine if changes in NDVI can be attributed to past and current grazing operations (Stone et al. 1992).

### Future Research

Our results suggest that there has been significant long-term browning of the Hawaiian Islands since 1982. Although we have identified landscapes and native ecosystems that have declined and that the overall climate of the Hawaiian Islands is becoming drier, there appear to be several individual and interacting causes for these declines. These include increased temperatures, fires, and changes in land use management (Lucas 2017). Indeed, the area burned annually by wildfire in Hawaii has increased fourfold in recent decades and is expected to continue to increase (Trauernicht 2019). Future research could examine 1) 250 m land cover and NDVI time series data from MODIS/VIIRS sensors to identify if these trends have occurred since 2000 along with impacts from active fire (1 km) and burned areas (500 m) on long term-trends (Giglio et al. 2018), and 2) monthly lags in native vegetation browning which was not considered in this study. Recently released 250 m climate data from 2000 to 2019 could also be used to identify associations with NDVI within pixels and land cover classes along with newly developed GIS maps of land management categories (Lucas 2017). This is especially needed for native vegetation types in the cloud zone of Hawaii, which contain a majority of Hawaii’s native biodiversity (Kagawa‐Viviani and Giambelluca 2020).

AVHRR NDVI annual and monthly time series data generated for this research along with change detections based on neutral phase years (e.g., 1984, 2001, 2019) can be processed and served via an interactive website (e.g., Global Forest Watch 2007 or ClimateEngine) to show long-term macro-temporal (e.g., two time periods based on neutral phase years and/or full-time series) and spatially explicit (5.1 km) trends of vegetation health and greenness back to 1982 (Bremer et al. 2015; GFW 2007). Indeed, it is not known if long-term declines in NDVI are occurring on other oceanic islands (e.g., Galapagos, New Caledonia, and Fiji) and this could be assessed using a similar AVHRR NDVI time series analysis.

## Conclusions

We used NDVI from AVHRR Climate Data Records products at 0.05 × 0.05° spatial resolution to identify significant long-term changes (1982 to 2019) in vegetation health for the Hawaiian Islands, each individual island, six land cover classes, and for native ecosystems. Overall, there has been a significant decline in NDVI on the Hawaiian Islands from 1982 to 2019 with Lāna’i and Hawai’i experiencing the greatest decreases in NDVI (≥44%). Developed, Agriculture, Bare Ground, Alpine/Subalpine, Non-Native, and Native classes significantly decreased in NDVI for most months, and this was especially true during the wet season month of March. The Native class on all islands experienced significant declines in NDVI, with the southwestern side of the island of Hawai’i experiencing the greatest declines in NDVI. The results show primarily positive correlations between the native ecosystem NDVI and precipitation, implying that significantly decreased precipitation may exacerbate the decrease in NDVI of the native ecosystems. The impacts of drought (PDSI) were more widespread than precipitation declines especially on the leeward side of Maui and Hawai’i and lower elevations of islands. NDVI-PDSI correlations in native ecosystems were primarily negative on the windward side and positive on the leeward side, suggesting a higher sensitivity to drought in leeward native ecosystems. Limitations include the large pixel size and cloud cover for higher elevation areas, which may impact the NDVI results. However, the near-forty-year time series and spatially explicit data for native landscapes can provide natural resource managers with long-term trends and monthly changes associated with vegetation health and stability. It is not known if these declines in NDVI will continue for native ecosystems in Hawaii or if similar trends are occurring on other oceanic islands.

## Supplementary Information

Supplementary Information

Supplementary Information
