# Supplementary figures and images for: A Near Four-Decade Time Series Shows the Hawaiian Islands Have Been Browning Since the 1980s
Source: Environ Manage. 2022 Nov 22;71(5):965–80. doi: 10.1007/s00267-022-01749-x (PMC10083158; doi:10.1007/s00267-022-01749-x)

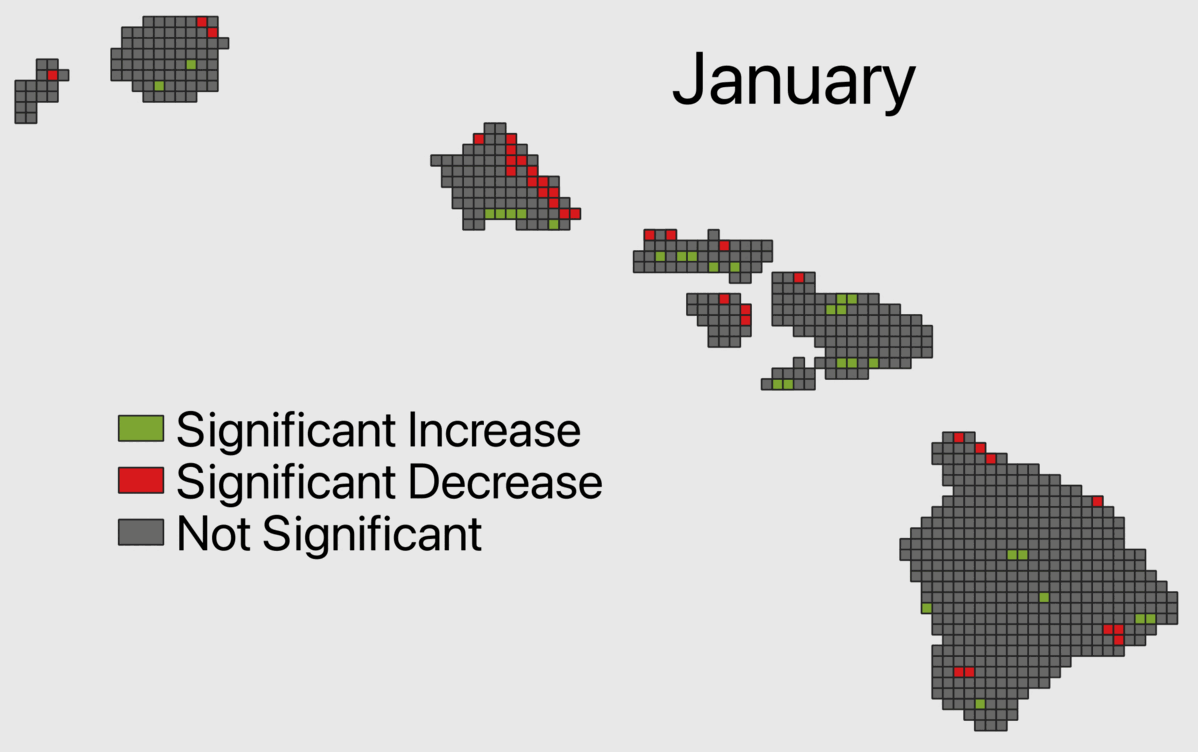

Supplement: Supplementary file 1 — Supplementary Information [file 267_2022_1749_MOESM1_ESM.gif]
