# Supplementary material for: A Near Four-Decade Time Series Shows the Hawaiian Islands Have Been Browning Since the 1980s
Source: Environ Manage. 2022 Nov 22;71(5):965–80. doi: 10.1007/s00267-022-01749-x (PMC10083158; doi:10.1007/s00267-022-01749-x)
Supplement: Supplementary file 2 — Supplementary Information [file 267_2022_1749_MOESM2_ESM.docx]

**A near four-decade time series shows the Hawaiian Islands have been browning since the 1980s**

Submitted to: *Environmental Management*

Austin Madson ^1*^, Monica Dimson ^2^, Lucas Berio Fortini ^3^, Kapua Kawelo ^4^, Tamara Ticktin ^5^, Matt Keir ^6^, Chunyu Dong ^7^, Zhimin Ma ^7^, David W. Beilman ^8^, Kelly Kay ^2^, Jonathan Pando Ocón ^2^, Erica Gallerani ^2^, Stephanie Pau ^9^, Thomas W. Gillespie ^2^

* Corresponding Author: Austin Madson, amadson@uwyo.edu

1 Wyoming Geographic Information Science Center, University of Wyoming, Laramie, WY, United States, amadson@uwyo.edu

2 Department of Geography, University of California Los Angeles, Los Angeles, CA, United States

3 U.S. Geological Survey, Pacific Island Ecosystems Research Center, Honolulu, HI, United States

4 Army Natural Resources Program, Schofield Barracks, HI, United States

5 School of Life Sciences, University of Hawaiʻi at Mānoa, Honolulu, HI, United States

6 Department of Land and Natural Resources, Division of Forestry and Wildlife, Honolulu, HI, United States

7 School of Civil Engineering, Sun Yat-sen University, Zhuhai, China

8 Department of Geography and Environment, University of Hawaiʻi at Mānoa, Honolulu, HI, United States

9 Department of Geography, Florida State University, Tallahassee, FL, United States

Figure SI 1. Trends in vegetation condition over the North Tropics from Advanced Very High Resolution Radiometer (AVHRR) data.

Figure SI 2. Time series of median Normalized Difference Vegetation Index (NDVI) for the purest land cover class (derived from the highest percentage of aggregated Carbon Assessment of Hawaii [CAH] land cover types) pixels based on Advanced Very High Resolution Radiometer (AVHRR) (0.05°) from 1982 to 2019.

Figure SI 3. Time series of AVHRR Normalized Difference Vegetation Index (NDVI) in Alpine/Subalpine classes along an elevational gradient (2763 m to 4023 m) on Mauna Loa, Island of Hawaiʻi, based on pixels with 100% Alpine/Subalpine landcover.

Figure SI 4. Changes in Normalized Difference Vegetation Index (NDVI) on Island of Hawaiʻi from this study (a) and environmental factors that could contribute to the significant declines in NDVI within the Native class on this island (b) forest cover change, (c) fire from GIS database, (d) burned areas from Moderate Resolution Imaging Spectroradiometer (MODIS), and (e) Rapid Ohia Death.

Figure SI 5. Time series of monthly median Normalized Difference Vegetation Index (NDVI) for two example tropical dry forests within the study area that can be used by resource managers to assess ecosystem health.

**Figure SI 1.** Trends in vegetation condition over the North Tropics from Advanced Very High Resolution Radiometer (AVHRR) data.

Monthly series of Vegetation Condition Index (VCI) from AVHRR on the left y axis and breakpoint location and piecewise trends for north tropical climate zone (0-23.5 N degrees) from 1981 to 2019 (Xue et al. 2020). An anomaly index is superimposed in red.


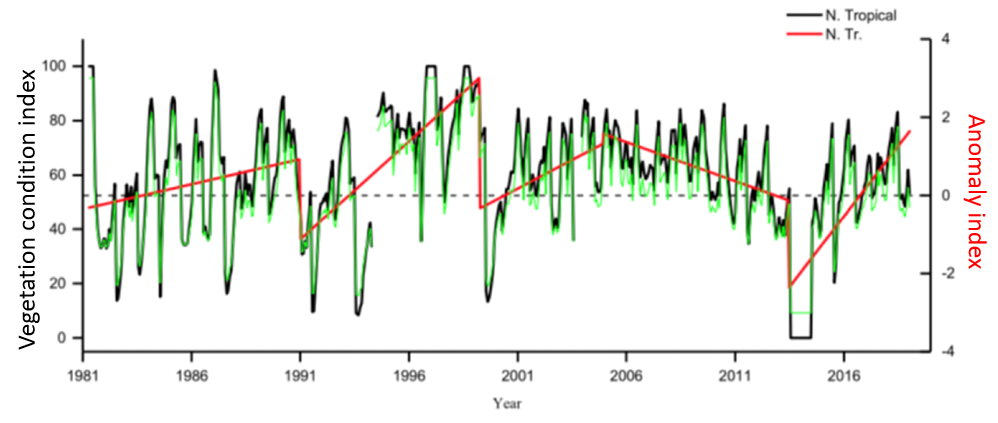


**Figure SI 2.** Time series of median Normalized Difference Vegetation Index (NDVI) for the purest land cover class (derived from the highest percentage of aggregated Carbon Assessment of Hawaii [CAH] land cover types) pixels based on Advanced Very High Resolution Radiometer (AVHRR) (0.05°) from 1982 to 2019.

Time series of pixels (5.1 by 5.1 km) with highest percentage land cover (e.g., 79% to 100%) for each aggregated land cover class in Carbon Assessment of Hawaiʻi (CAH) (Jacobi et al. 2017). Several macro-trends were identified across the time series. Declines in Normalized Difference Vegetation Index (NDVI) were pronounced in 1989 for all land cover classes, which corresponds to the 1989 La Niña event. For Developed areas in Honolulu, Oʻahu (a) and Agriculture areas near Pā'ia, Maui (b), declines were pronounced in 1989, 1995, 2001, 2010, and 2019. Bare ground in Volcanoes National Park, Hawaiʻi (c) and Alpine/Subalpine areas on Mauna Loa (3783 m), Hawaiʻi (d) showed similar trends. However, after 2017, NDVI decreased for the Bare Ground class in Volcanoes National Park while Alpine/Subalpine class increased on Mauna Loa (3783 m). The Non-Native class southeast of Waimea on Hawai'i (e) was primarily *Leucaena leucocephala* and the Native class (f) (native lowland rain forest in Paauilo, Hawaiʻi) both showed similar trends with pronounced declines in 1989 and 2019.

a) Developed: Honolulu, O'ahu


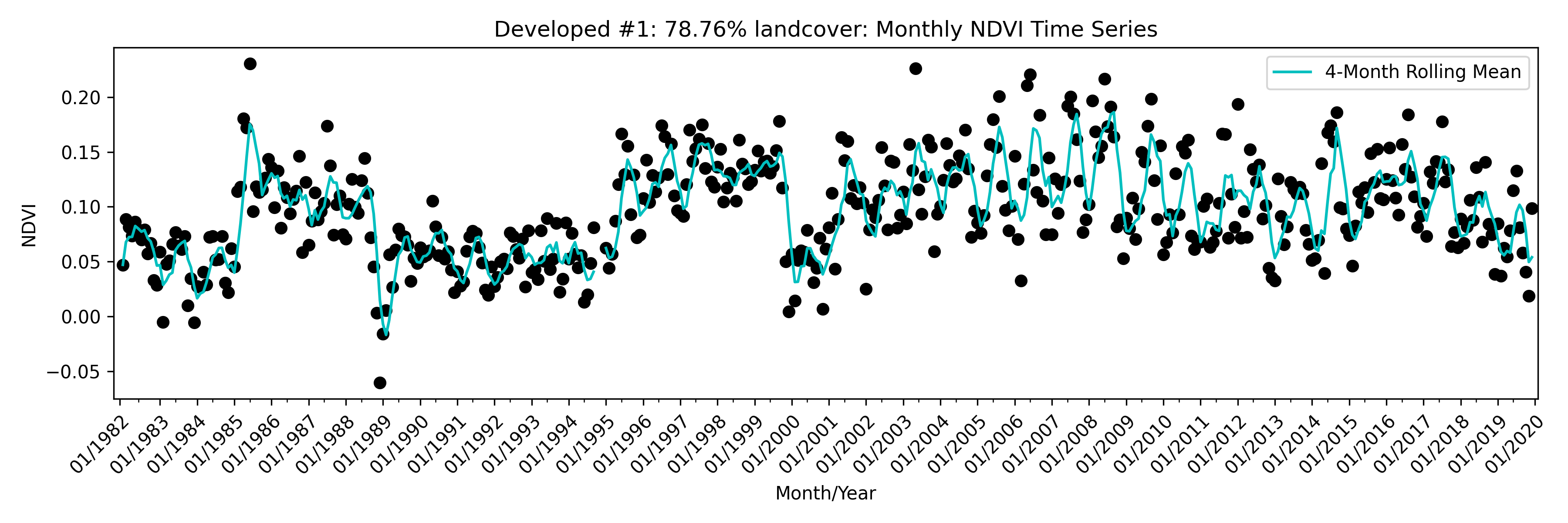


b) Agriculture: Pā'ia, Maui


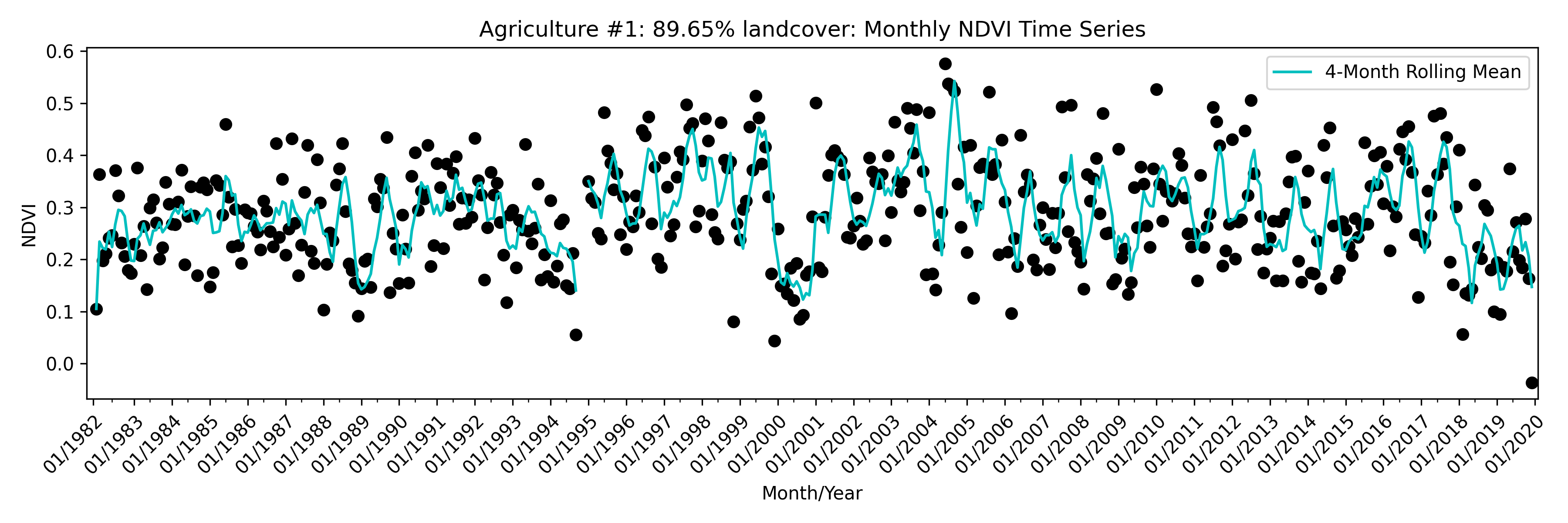


c) Bare Ground: Volcanoes National Park, Hawai'i


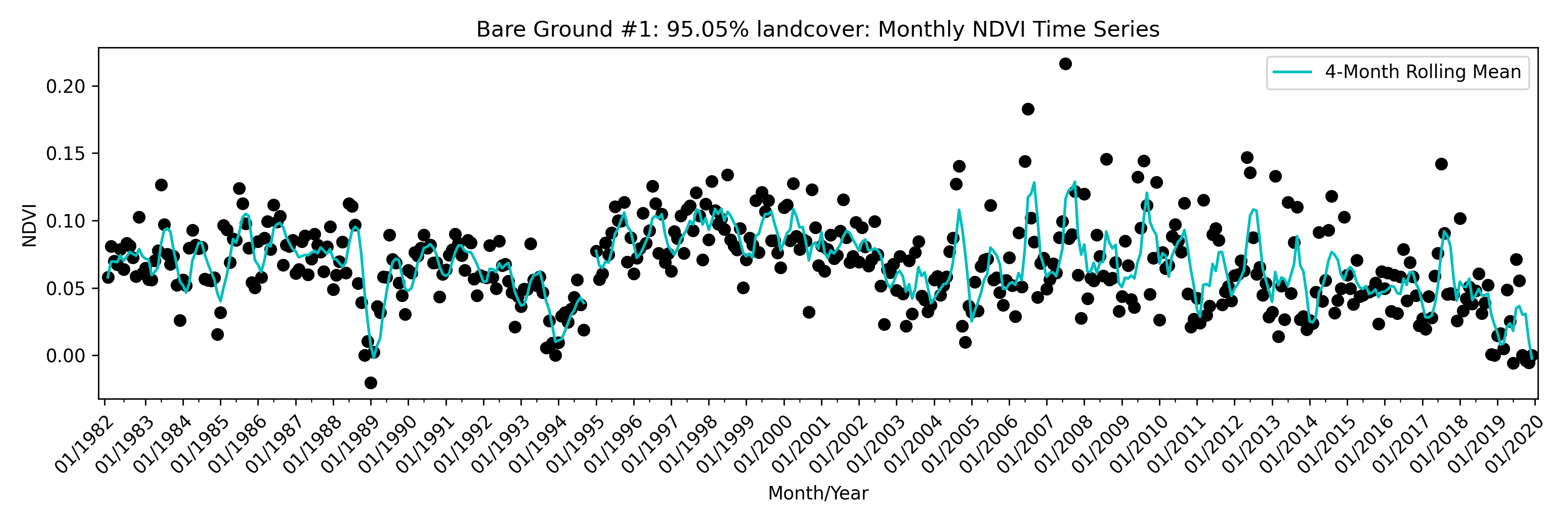


d) Alpine/Subalpine: Mauna Loa (3783 m), Hawai'i


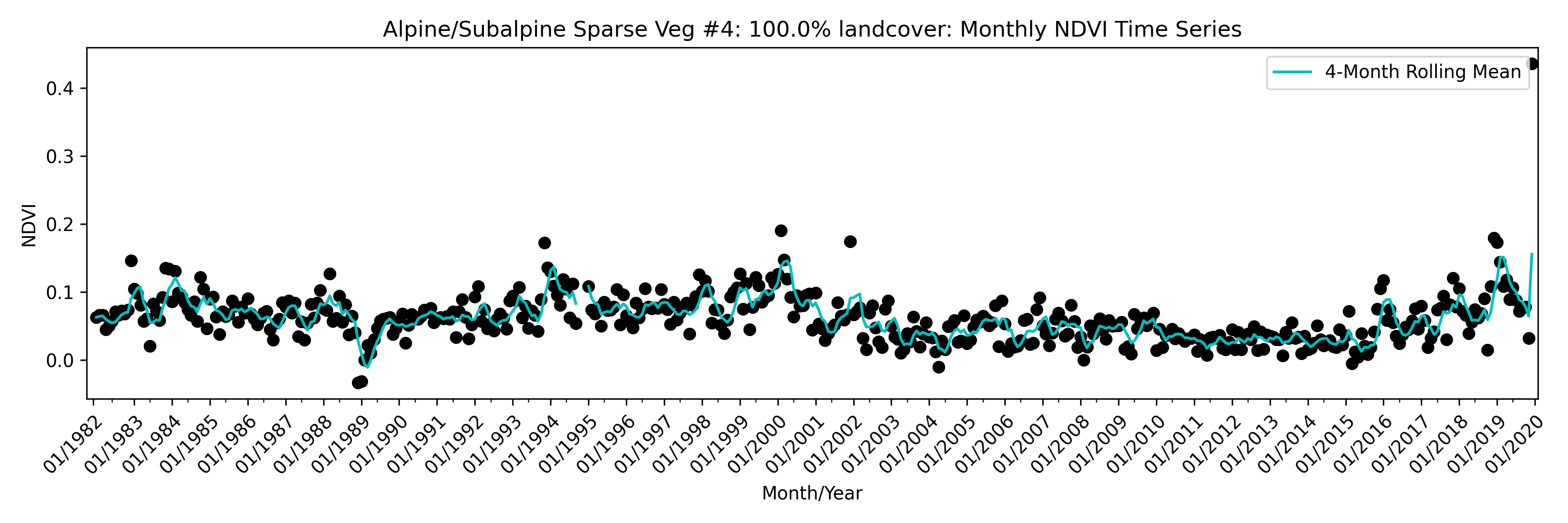


e) Non-Native: southeast of Waimea on Hawai'i


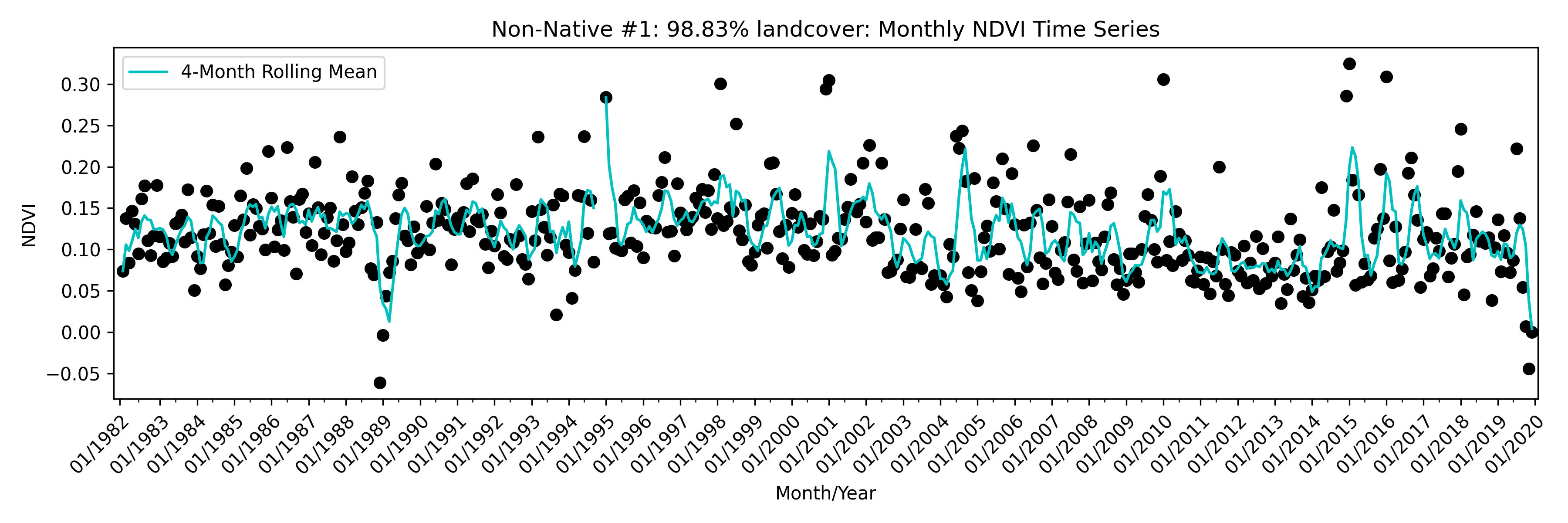


f) Native: Paauilo, Hawai'i


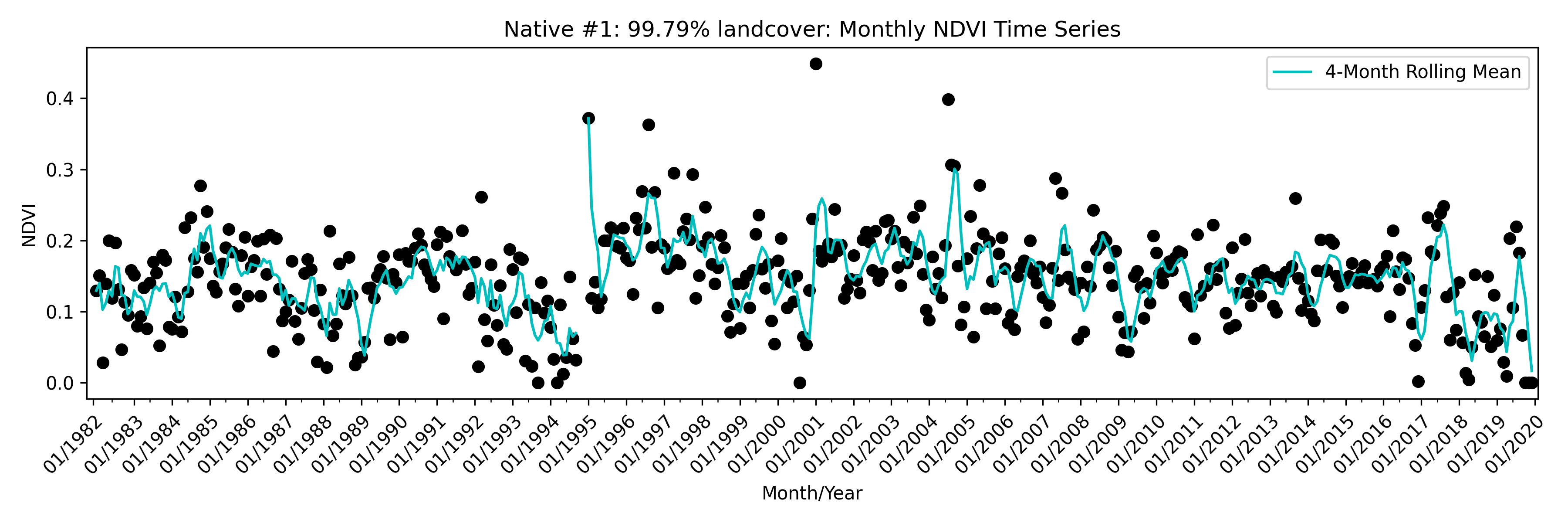


Figure SI 3. Time series of AVHRR Normalized Difference Vegetation Index (NDVI) in Alpine/Subalpine classes along an elevational gradient (2763 m to 4023 m) on Mauna Loa, Island of Hawaiʻi, based on pixels with 100% Alpine/Subalpine landcover.

**
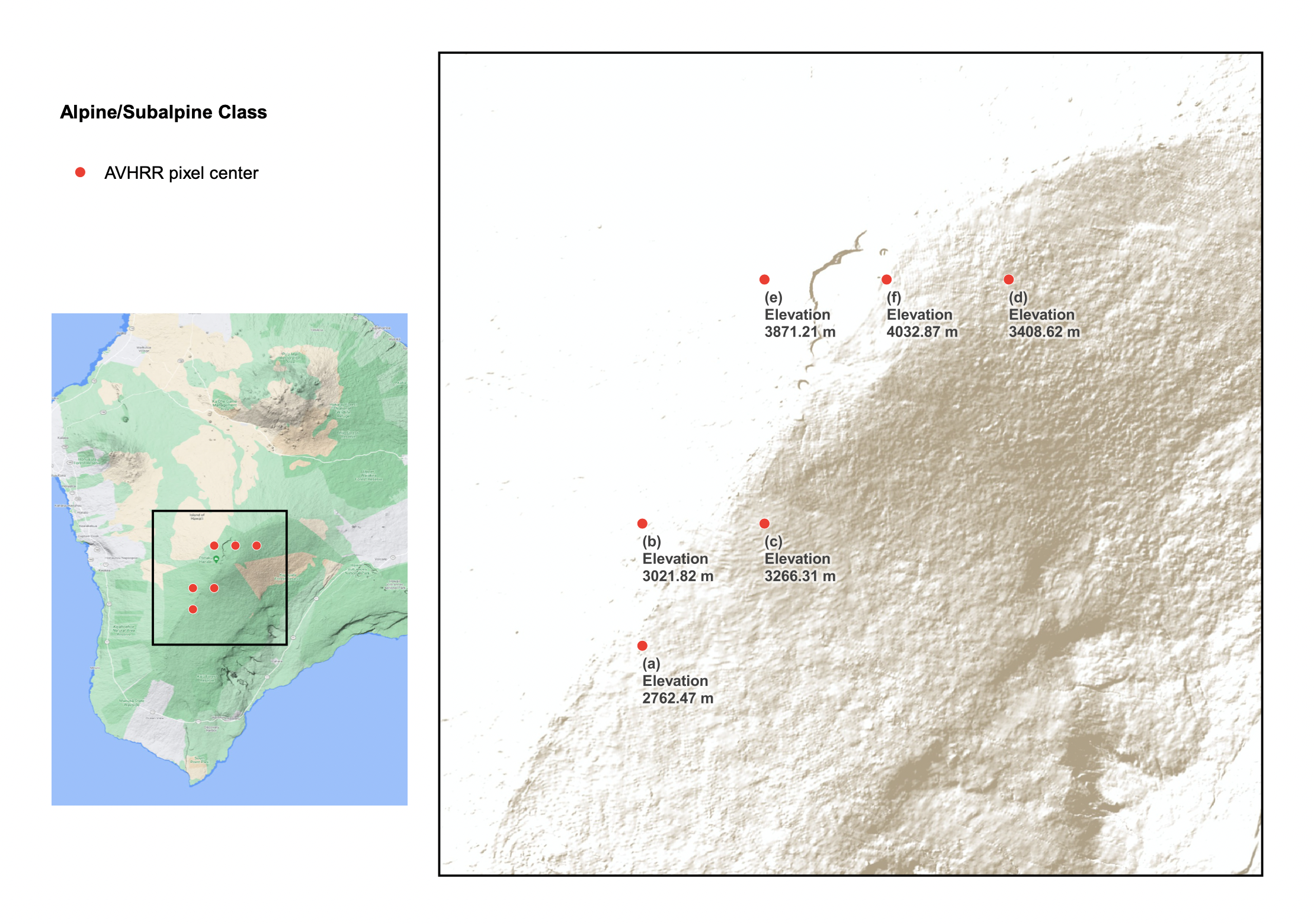
**

a) Elevation 2763 m


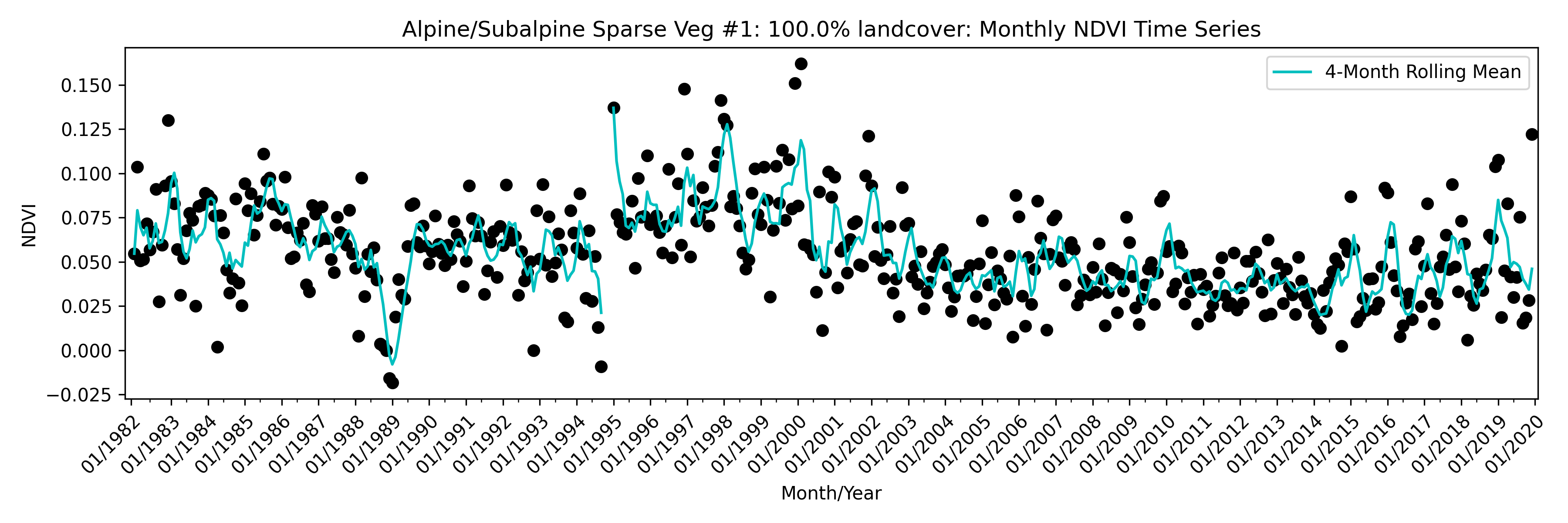


b) Elevation 3024 m


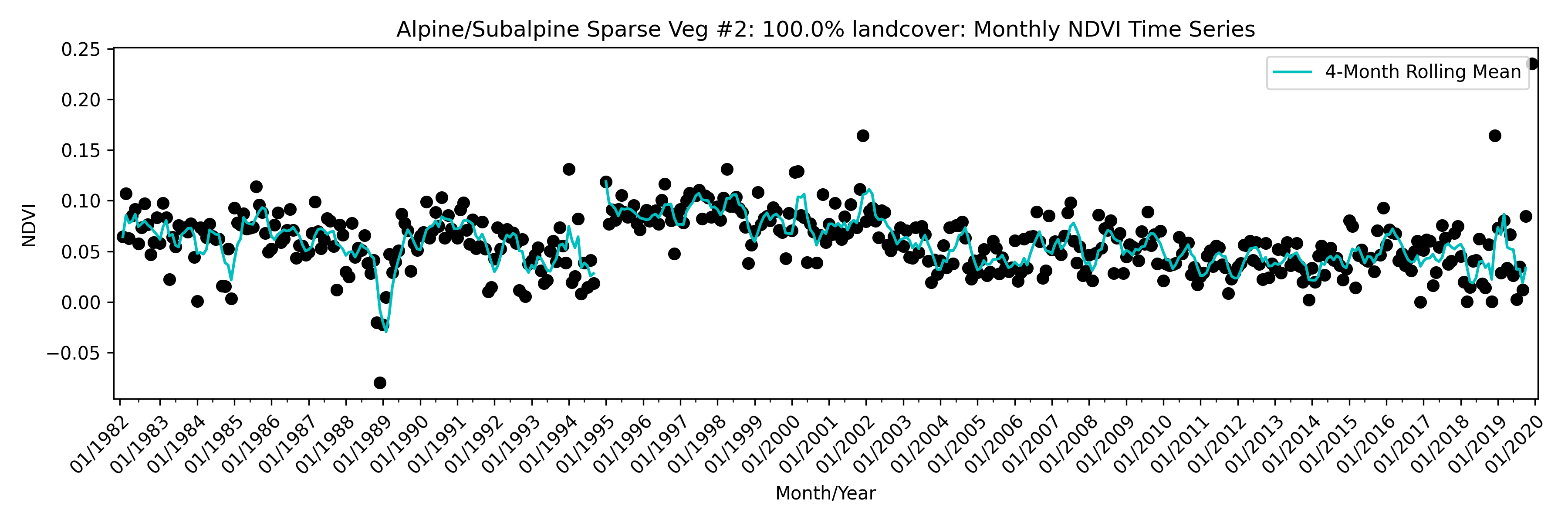


c) Elevation 3273 m


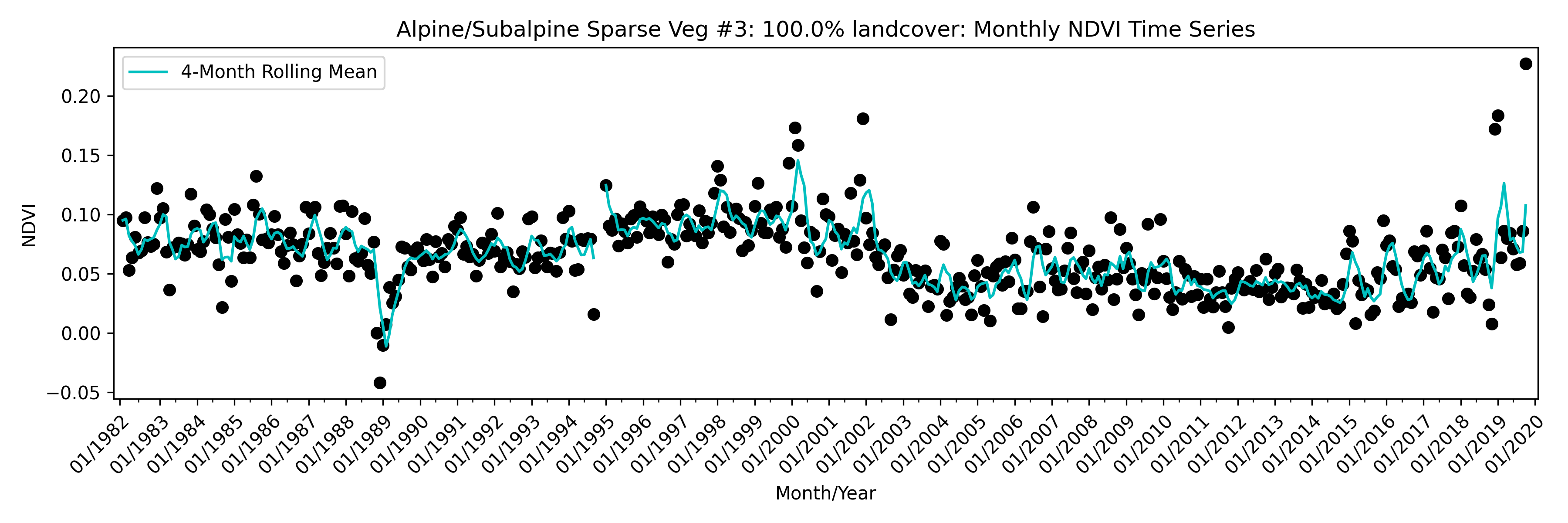


d) Elevation 3422 m


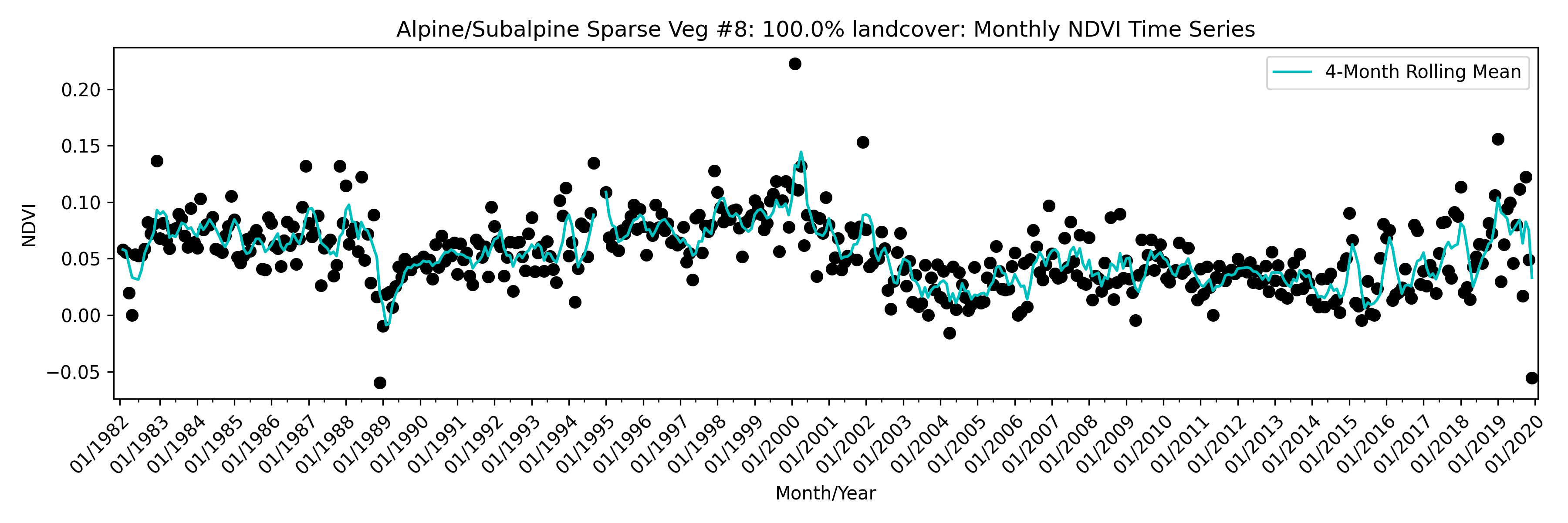


e) Elevation 3882 m


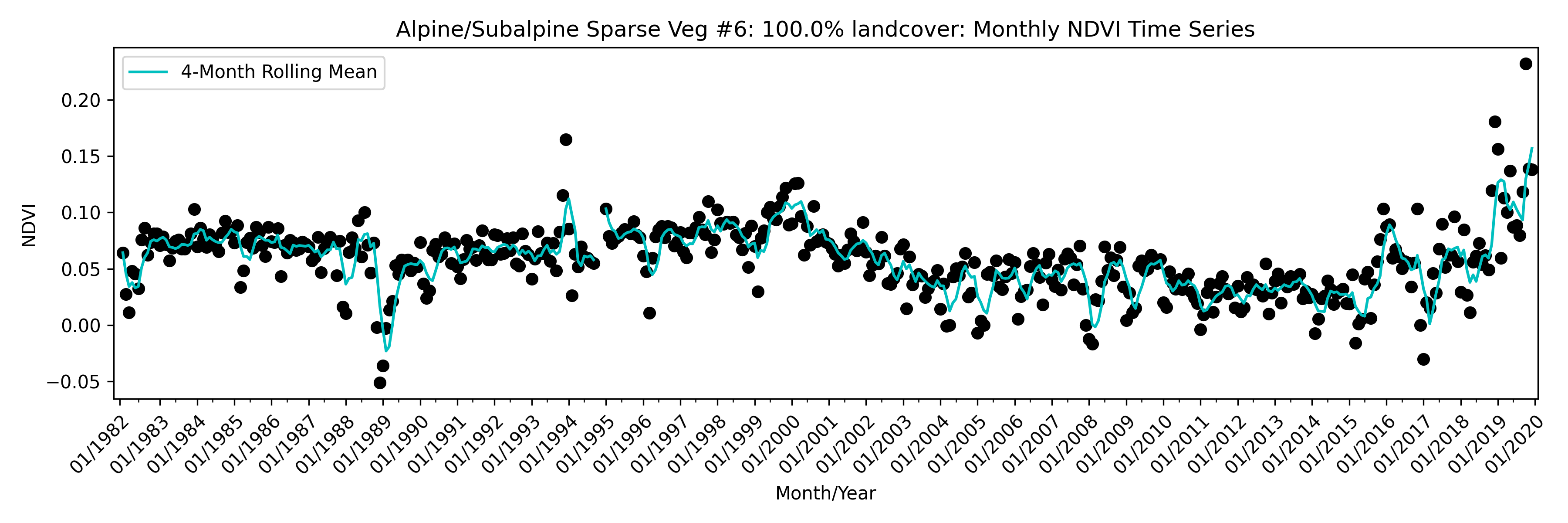


f) Elevation 4023 m


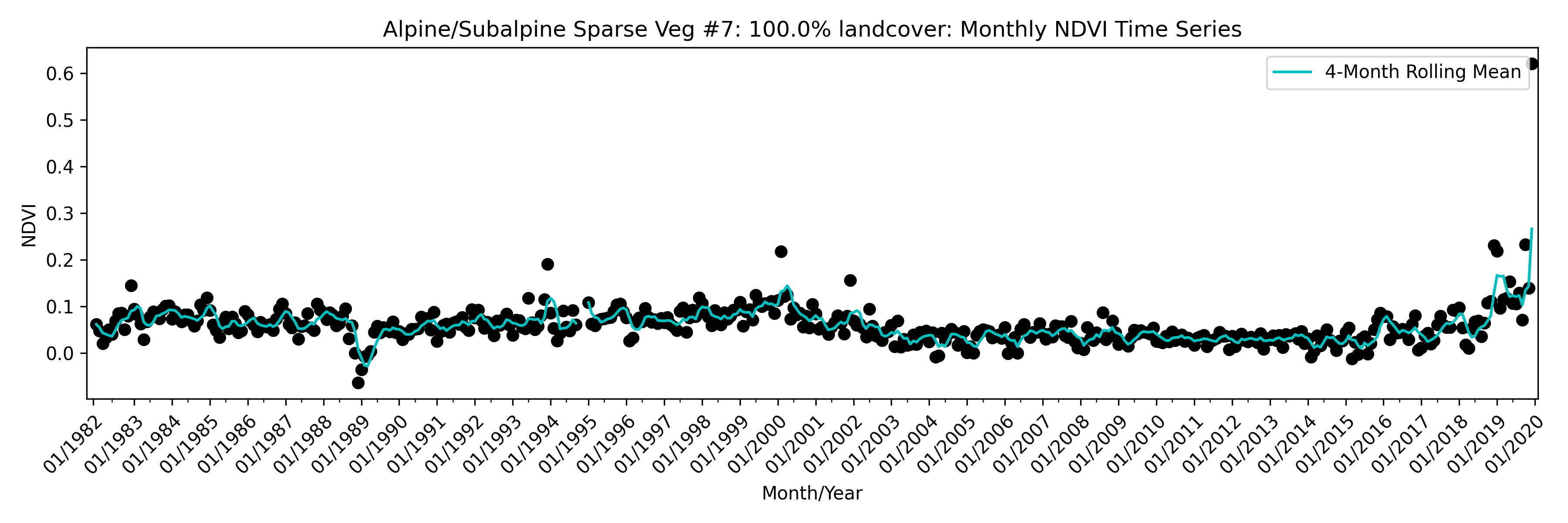


Figure SI 4. Changes in Normalized Difference Vegetation Index (NDVI) on Island of Hawaiʻi from this study (a) and environmental factors that could contribute to the significant declines in NDVI within the Native class on this island (b) forest cover change, (c) fire from GIS database, (d) burned areas from Moderate Resolution Imaging Spectroradiometer (MODIS), and (e) Rapid Ohia Death.

Figure SI 4a. Significant changes in Normalized Difference Vegetation Index (NDVI) from 1982 to 2019 on the Island of Hawaiʻi from this study.


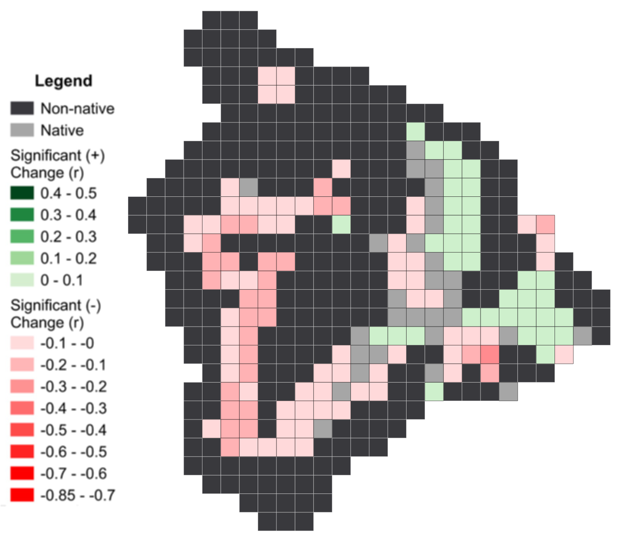


Figure SI 4b. Forest cover change on the Island of Hawaiʻi. Data from Hansen et al. (2013)


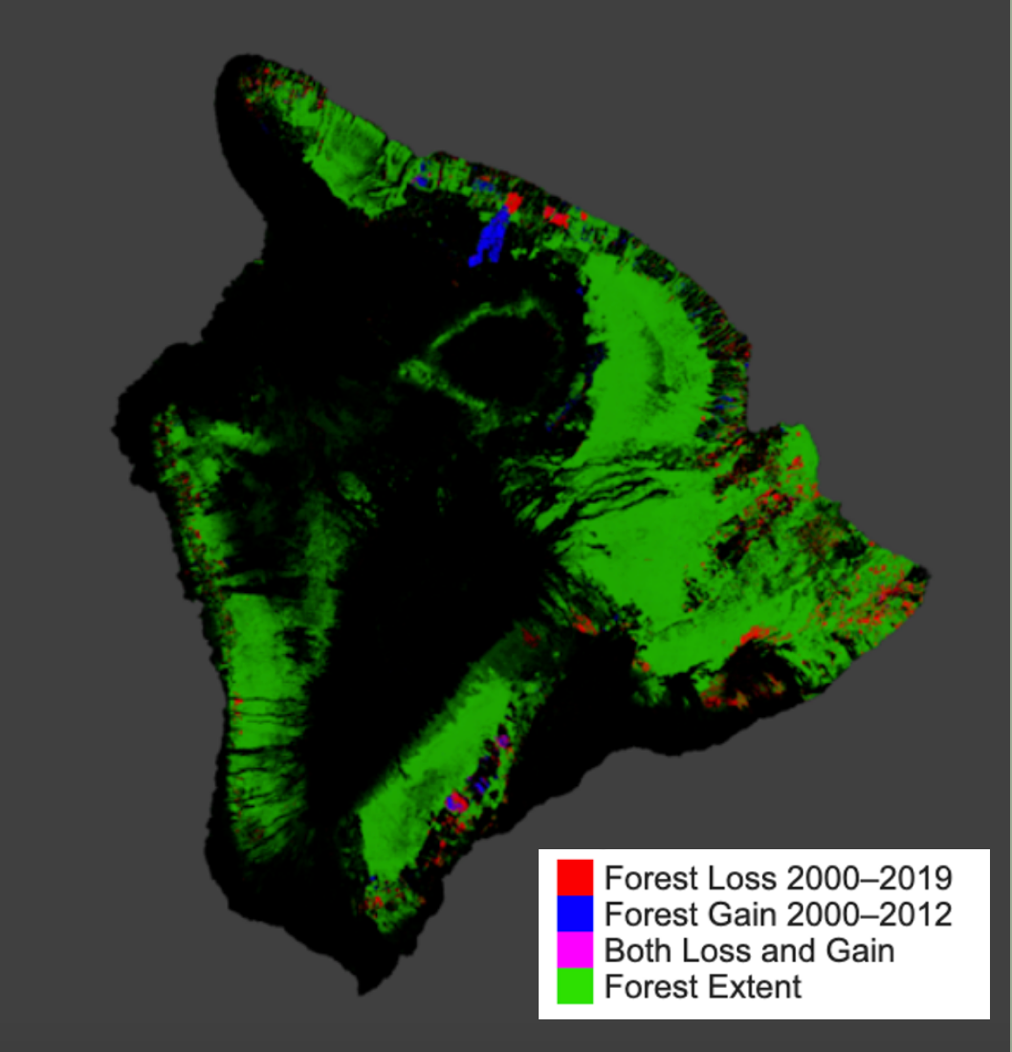


Figure SI 4c. Fire history (area and frequency) on the Hawaiian Islands. Fire data are from the Hawaiʻi State Wildfire History Dataset. The Hawai'i State Wildfire History Dataset was compiled by the Hawai'i Wildfire Management Organization and includes data from each of the state’s fire response agencies (excluding the Department of Defense) (Hawaii Wildfire Management Organization, 2013).


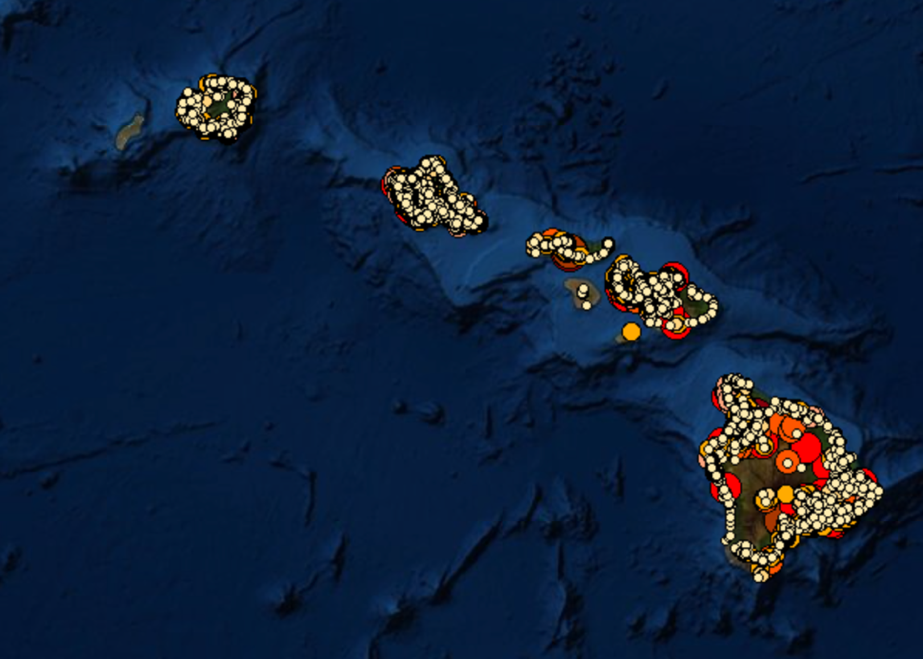


Figure SI 4d. Fires occurring in Native pixels from 2000 to 2020 on the Island of Hawaiʻi. Burned area (red) in Native pixels from 2000 to 2020 from Moderate Resolution Imaging Spectroradiometer (MODIS) MCD64A1 (Burned Area) products. The total burned area from 2000 to 2020 covered 46.25 km^2^ (0.826%) of Native pixels on the island of Hawaiʻi. The Terra and Aqua combined MCD64A1 Version 6 Burned Area data product is a monthly, global gridded 500-m product containing per-pixel burned-area and quality information. The MCD64A1 burned-area mapping approach uses 500-m MODIS Surface Reflectance imagery coupled with 1-km MODIS active fire observations. The algorithm uses a burn sensitive vegetation index (VI) to create dynamic thresholds that are applied to the composite data. The VI is derived from MODIS shortwave infrared atmospherically corrected surface reflectance bands 5 and 7 with a measure of temporal texture. The algorithm identifies the date of burn for the 500- by 500-m grid cells within each individual MODIS tile. The date is encoded in a single data layer as the ordinal day of the calendar year on which the burn occurred, with values assigned to unburned land pixels and additional special values reserved for missing data and water grid cells.


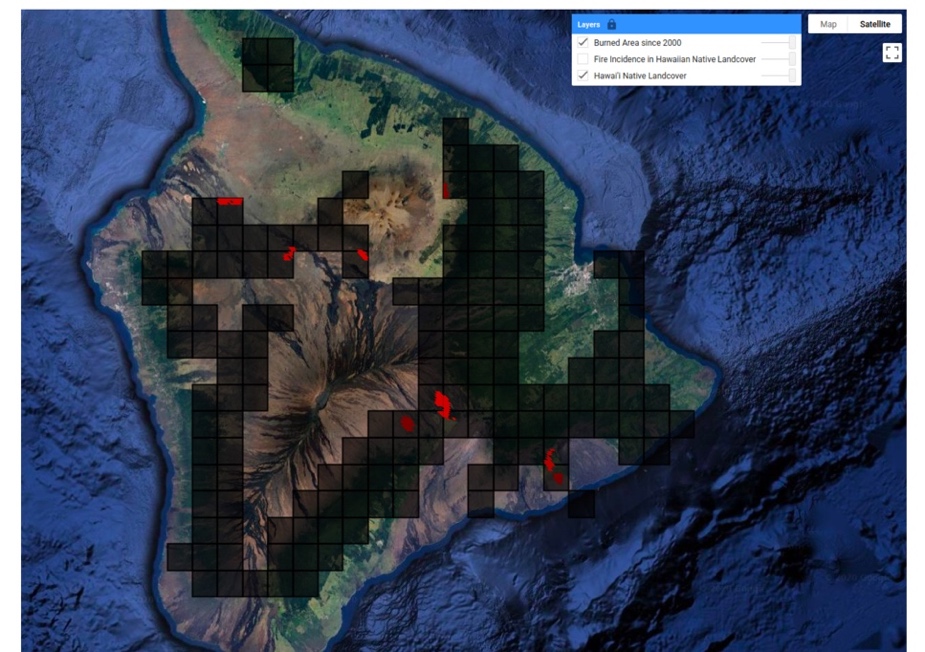


Figure SI 4e. Rapid Ohia Death (*Ceratocystis lukuohia* and *Ceratocystis huliohia*) on the Island of Hawaiʻi. Data are from the College of Tropical Agriculture and Human Resources (UH Manoa), 2020.


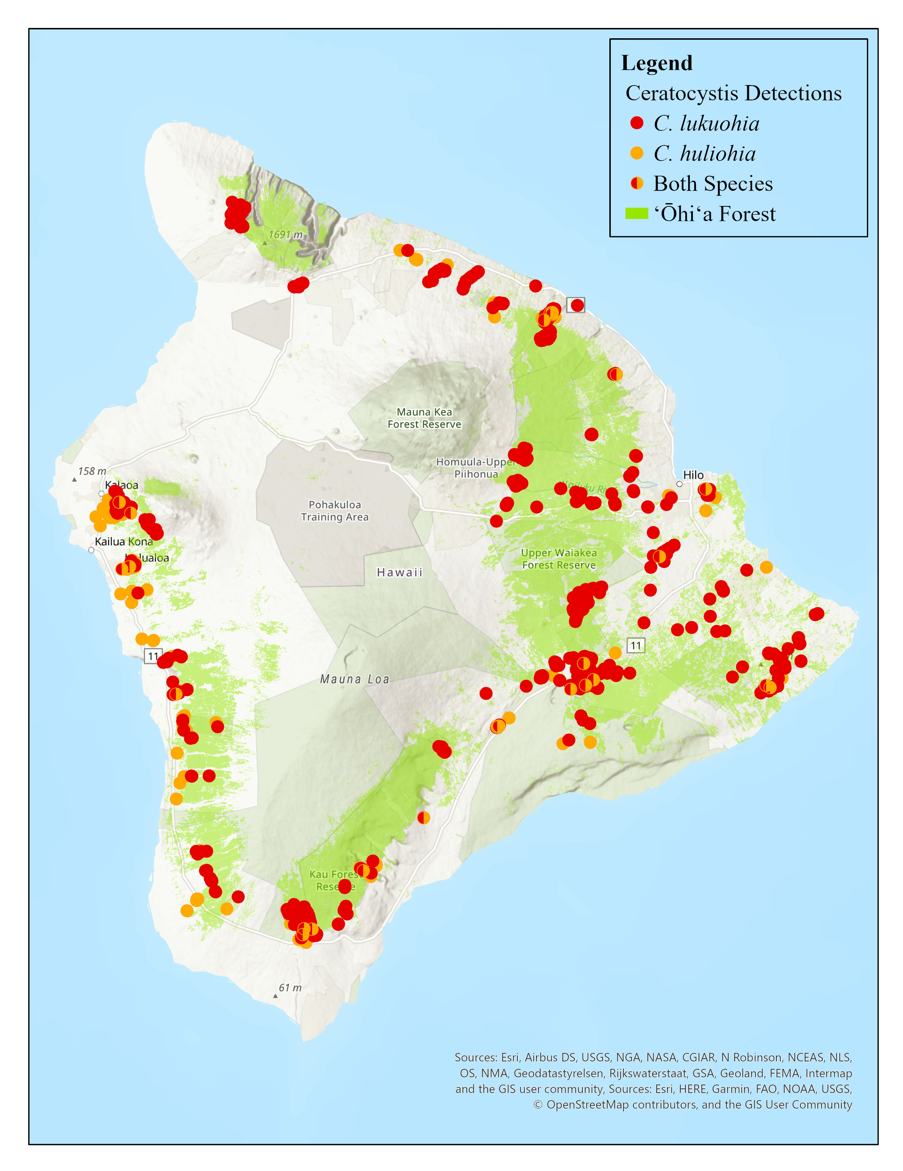


Figure SI 5. Example of Normalized Difference Vegetation Index (NDVI) time series from Advanced Very High Resolution Radiometer (AVHRR) for 1982 to 2019 and monthly change with significant changes based on linear regressions that can be used by natural resource managers to assess ecosystem health: (a) Kaʿūpūlehu (Island of Hawaiʻi) and (b) Auwahi (Island of Maui) dry forests.

Figure SI 5a. Kaupulehu


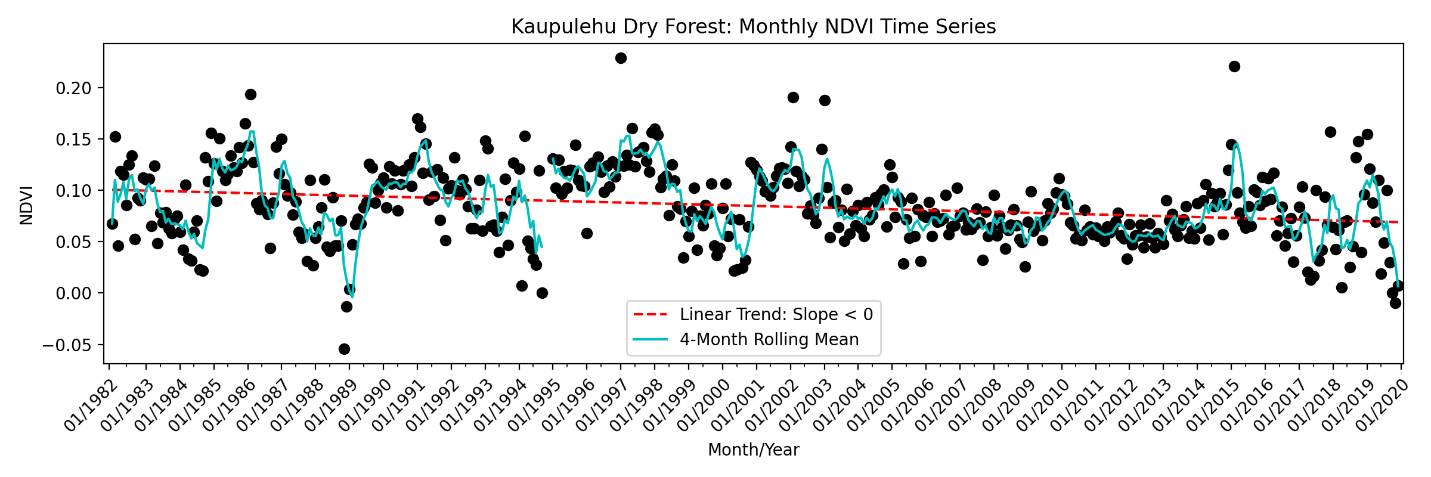


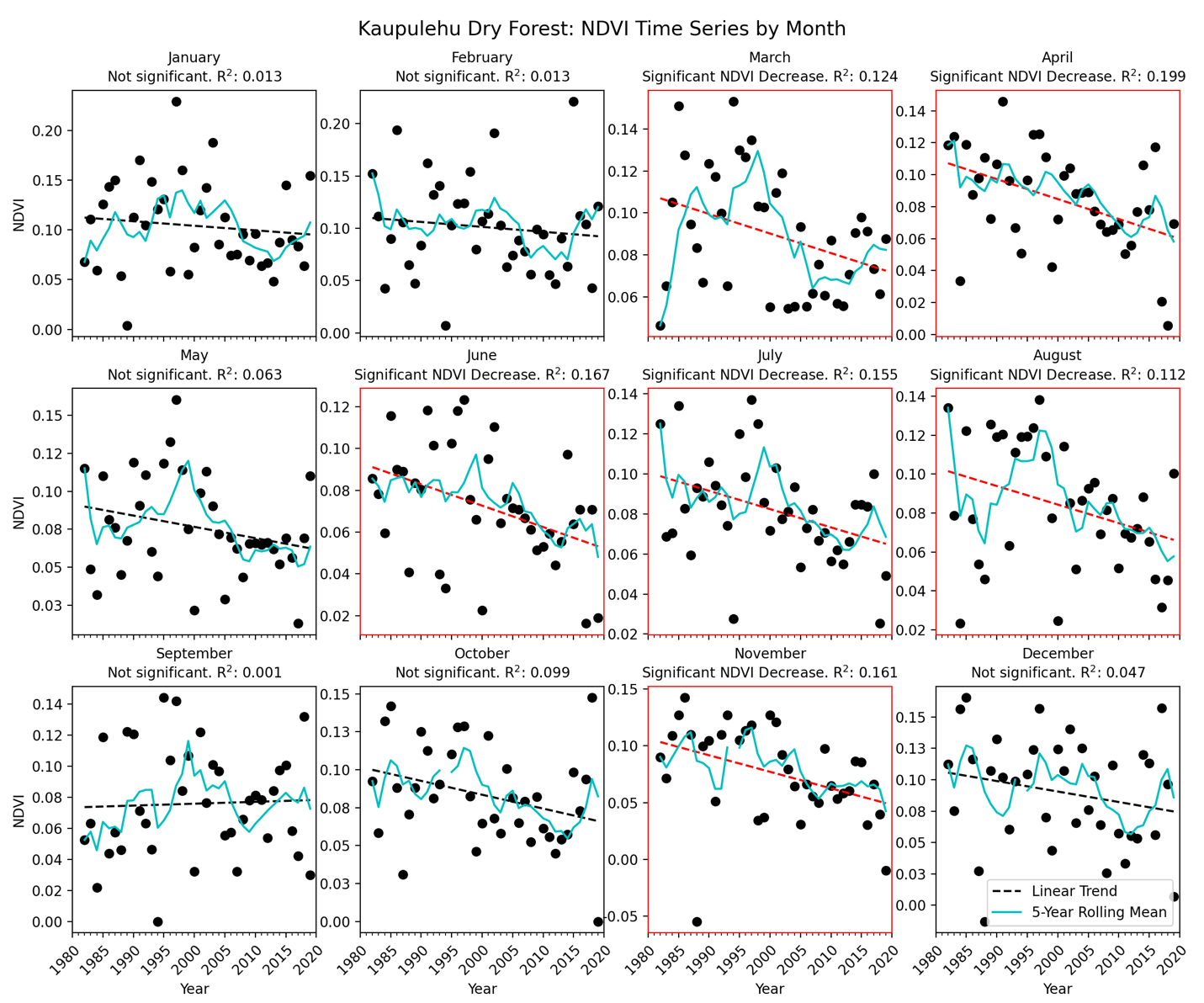


Figure SI 5b. Auwahi dry forests


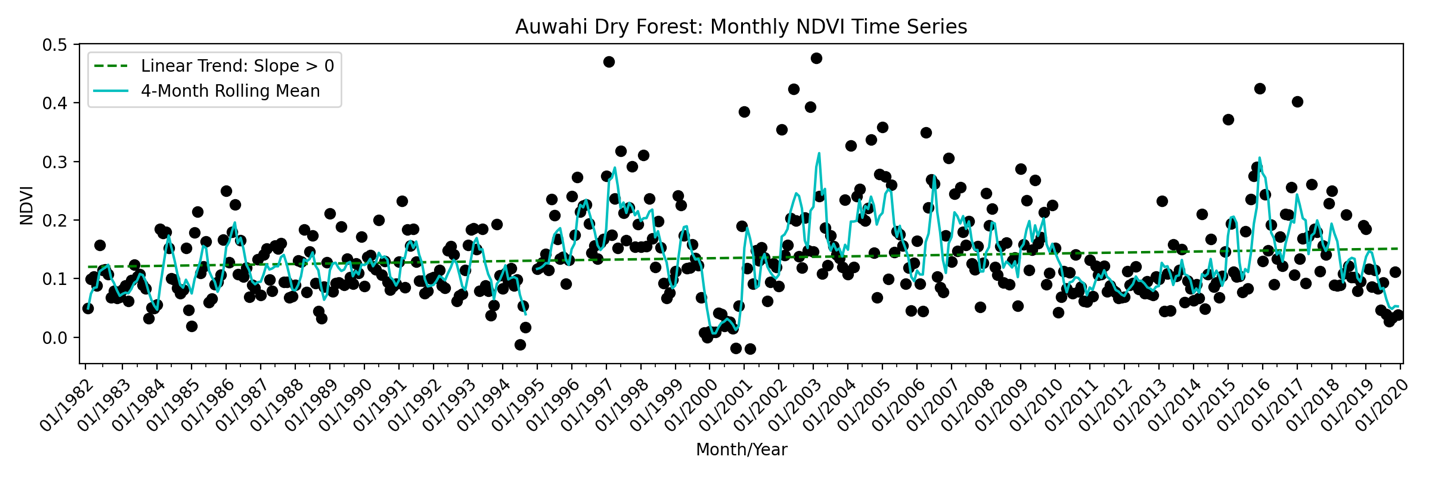


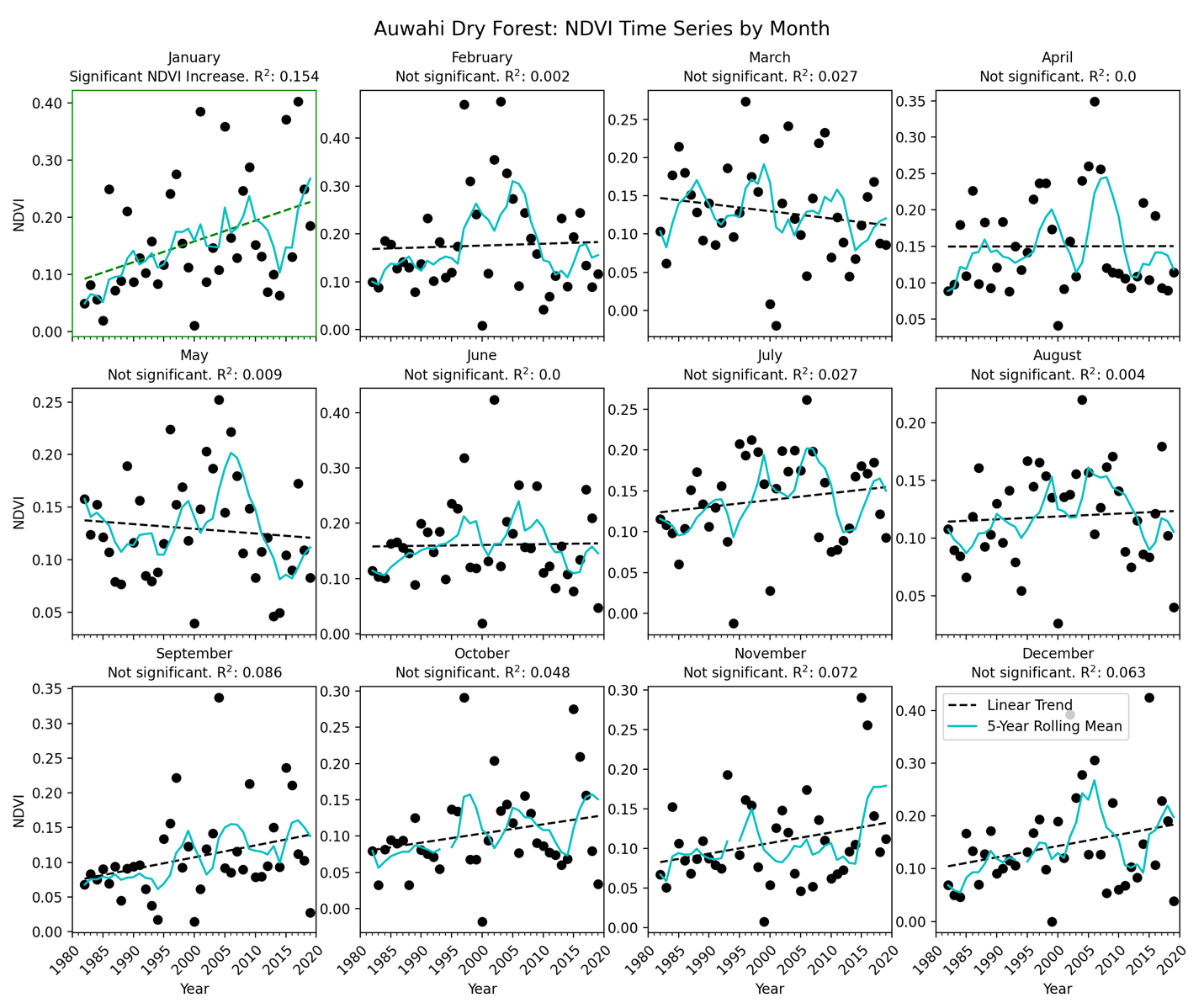


Any use of trade, firm, or product names is for descriptive purposes only and does not imply endorsement by the U.S. Government.

**References**

Hansen MC, Potapov PV, Moore R, Hancher M, Turubanova SA, Tyukavina A, Thau D, Stehman SV, Goetz SJ, Loveland TR, Kommareddy A, Egorov A, Chini L, Justice CO, Townshend JRG (2013). High-resolution global maps of 21st- century forest cover change. Science 342:850-853. <https://doi.org/10.1126/science.1244693>

Hawaii Wildfire Management Organization. 2013. Hawaii State Wildfire History Data Set. Available online at <https://gis.ctahr.hawaii.edu>, <https://www.hawaiiwildfire.org/>

Jacobi JD, Price JP, Fortini LB, Gon III SM, Berkowitz P (2017) Baseline land cover. In Baseline and projected future carbon storage and carbon fluxes in ecosystems of Hawai‘i, U.S. Geological Survey Professional Paper 1834, <https://doi.org/10.3133/pp1834>.

UH Manoa - College of Tropical Agriculture and Human Resources, June 2020. Rapid Ohia Death. Accessed on November 1, 2022. <https://cms.ctahr.hawaii.edu/rod/THE-DISEASE/DISTRIBUTION>

Xu Z, Cao L, Zhong S, Liu G, Yang Y, Zhu S, Luo X, Di L (2020) Trends in global vegetative drought from long-term satellite remote sensing data. IEEE J Sel Top Appl Earth Obs Remote Sens 13:815–826. <https://doi.org/10.1109/JSTARS.2020.2972574>
